# PD-L1 Overexpression, SWI/SNF Complex Deregulation, and Profound Transcriptomic Changes Characterize Cancer-Dependent Exhaustion of Persistently Activated CD4^+^ T Cells

**DOI:** 10.3390/cancers13164148

**Published:** 2021-08-18

**Authors:** Iga Jancewicz, Joanna Szarkowska, Ryszard Konopinski, Malgorzata Stachowiak, Monika Swiatek, Katarzyna Blachnio, Szymon Kubala, Paulina Oksinska, Pawel Cwiek, Natalia Rusetska, Agnieszka Tupalska, Natalia Zeber-Lubecka, Ewa Grabowska, Bianka Swiderska, Agata Malinowska, Michal Mikula, Beata Jagielska, Jan Walewski, Janusz A. Siedlecki, Tomasz J. Sarnowski, Sergiusz Markowicz, Elzbieta A. Sarnowska

**Affiliations:** 1 Department of Experimental Immunotherapy, Maria Sklodowska-Curie National Research Institute of Oncology, 02-781 Warsaw, Poland; iga.jancewicz@pib-nio.pl (I.J.); joanna.szarkowska@pib-nio.pl (J.S.); ryszard.konopinski@pib-nio.pl (R.K.); malgorzata.stachowiak@pib-nio.pl (M.S.); monika.swiatek@pib-nio.pl (M.S.); agnieszka.tupalska@unibas.ch (A.T.); ewa.grabowska@pib-nio.pl (E.G.); sergiusz.markowicz@pib-nio.pl (S.M.); 2 Department of Pathology, Maria Sklodowska-Curie National Research Institute of Oncology, 02-781 Warsaw, Poland; katarzyna.blachnio@pib-nio.pl; 3 Institute of Biochemistry and Biophysics, Polish Academy of Sciences, 02-106 Warsaw, Poland; szymon.globus@ibb.waw.pl (S.K.); p.oksinska@ibb.waw.pl (P.O.); p.cwiek@ibb.waw.pl (P.C.); bianka.swiderska@ibb.waw.pl (B.S.); esme@ibb.waw.pl (A.M.); 4 Department of Molecular and Translational Oncology, Maria Sklodowska-Curie National Research Institute of Oncology, 02-781 Warsaw, Poland; natalia.rusetska@pib-nio.pl (N.R.); janusz.siedlecki@pib-nio.pl (J.A.S.); 5 Department of Biomedicine, University Hospital Basel and University of Basel, 4001 Basel, Switzerland; 6 Department of Genetics, Maria Sklodowska-Curie National Research Institute of Oncology, 02-781 Warsaw, Poland; natalia.zeber-lubecka@cmkp.edu.pl (N.Z.-L.); michal.mikula@pib-nio.pl (M.M.); 7 Department of Gastroenterology, Hepatology and Clinical Oncology, Centre of Postgraduate Medical Education, 01-826 Warsaw, Poland; 8 Department of Oncology and Internal Medicine, Maria Sklodowska-Curie National Research Institute of Oncology, 02-781 Warsaw, Poland; beata.jagielska@pib-nio.pl; 9 Department of Lymphoid Malignancies, Maria Sklodowska-Curie National Research Institute of Oncology, 02-781 Warsaw, Poland; jan.walewski@pib-nio.pl

**Keywords:** CD4^+^ effector T cells, T cell exhaustion, SWI/SNF complex, PD-1, PD-L1, PD-L1/PD-1 axis

## Abstract

**Simple Summary:**

Growing tumors induce an immune response. For proper immune response, both CD8^+^ and CD4^+^ effector T cells are required. Tumors avoid attacks from tumor-infiltrating lymphocytes (TILs) via induction of several inhibitory signals, such as PD-L1/2, which bind to the PD-1 receptor, consequently leading to T cell dysfunction, exhaustion, and apoptosis. The mechanism of T cell exhaustion has been studied mostly in CD8+ T cells, although some results suggest that CD4^+^ effector T cells also undergo exhaustion. In this study, we analyze global transcript profiling, PD-1 and PD-L1 expression, and chromatin status on the PD-L1 locus. We find that in exhausted CD4^+^ T cells, the levels of PD-L1 are increased at both the transcript and protein levels, while PD-L1 expression depends on SWI/SNF chromatin remodeling and PRC2-repressive complexes. The expression of PD-L1 in exhausted CD4^+^ T cells can be reversible.

**Abstract:**

Growing tumors avoid recognition and destruction by the immune system. During continuous stimulation of tumor-infiltrating lymphocytes (TILs) by tumors, TILs become functionally exhausted; thus, they become unable to kill tumor cells and to produce certain cytokines and lose their ability to proliferate. This collectively results in the immune escape of cancer cells. Here, we show that breast cancer cells expressing PD-L1 can accelerate exhaustion of persistently activated human effector CD4^+^ T cells, manifesting in high PD-1 and PD-L1 expression level son T cell surfaces, decreased glucose metabolism genes, strong downregulation of SWI/SNF chromatin remodeling complex subunits, and p21 cell cycle inhibitor upregulation. This results in inhibition of T cell proliferation and reduction of T cell numbers. The RNAseq analysis on exhausted CD4^+^ T cells indicated strong overexpression of IDO1 and genes encoding pro-inflammatory cytokines and chemokines. Some interleukins were also detected in media from CD4^+^ T cells co-cultured with cancer cells. The PD-L1 overexpression was also observed in CD4^+^ T cells after co-cultivation with other cell lines overexpressing PD-L1, which suggested the existence of a general mechanism of CD4^+^ T cell exhaustion induced by cancer cells. The ChIP analysis on the PD-L1 promoter region indicated that the BRM recruitment in control CD4^+^ T cells was replaced by BRG1 and EZH2 in CD4^+^ T cells strongly exhausted by cancer cells. These findings suggest that epi-drugs such as EZH2 inhibitors may be used as immunomodulators in cancer treatment.

## 1. Introduction

In developed tumors, the cancer cells avoid immune response executed by tumor-infiltrating T cells (tumor-infiltrating lymphocytes—TILs) [1]. T cell exhaustion occurs when T cells are unable to perform their function and become dysfunctional. This phenomenon occurs during chronic infections and cancers [2]. The exhaustion process is linear and defined as a loss of many effector functions by T cells, expression of multiple inhibitory receptors, and altered transcription profile [3]. Collectively, the term ‘T cell exhaustion’ describes a broad spectrum of T cell responses to chronic antigen stimulation due to viral infection and T cell responses to tumors; however, the exact mechanism behind this process still remains elusive. As such, deciphering of the mechanism of T lymphocyte exhaustion is crucial for the development of successful checkpoint blockade immunotherapies and adoptive T cell therapies [4]. Modern immunotherapy with autologous CAR-T cells is a new and successful approach to treat hematological malignancies, although it has some limitations. The CAR-T cells from non-responding patients have upregulated exhaustion and apoptosis markers [5]. In fact, exhaustion is a dynamic process ranging from altered functionality of T cells to complete lack of their effector function. CD8^+^ T cells are still perceived as a standard cytotoxic immune response against cancer; therefore, the majority of research concerning exhaustion has been conducted on effector CD8^+^ T cells. As the specific hallmarks for exhaustion, ample genes with characteristic functions were found to be overexpressed in CD8^+^ T cells. In particular, genes encoding several transcription factors, e.g., YY1, FOXO1, NFAT1, BLIMP1, IRF4, BATF, and Eomes, as well as checkpoint receptors such as PD1, Lag3, Tim3, and TIGIT, were overexpressed in CD8^+^ T cells that underwent exhaustion. In parallel, the downregulation of the cytokine axis, i.e., IL2, IFNγ, and TNFα, was observed in these cells [6]. Infiltrating tumor T cells experience hypoxia leading to the induction of mitochondrial stress, and consequently to rapid exhaustion [7]. Effector T cell exhaustion is directed by a global interplay between networks of transcription factors, coactivators, and corepressors, which are often involved in synergistic or mutually antagonistic pathways [8]. Effector and memory T cells exhibit similar patterns and programs of transcriptional regulators, while T cells that have undergone exhaustion are characterized by entirely distinct patterns of epigenetic modification and chromatin accessibility with a state-specific epigenetic landscape. During exhaustion process, global changes in epigenetic landscape are observed. Epigenetic signatures of exhausted T cells are determined by the analysis of histone H3 acetylation (permissive marker) and trimethylation (repressive marker) on K9 and K27 positions, respectively. Additionally, high expression of *DNMT1* (DNA methyltransferase 1), *MT3B* (methyltransferase 3B), and *EZH2* (enhancer of Zeste homolog 2), which is the main subunit of polycomb-repressive complex 2 (PRC2) [8], is observed in exhausted T cells. Interestingly, the T cell exhaustion may also be mediated by metabolic changes in the local environment. The microenvironment for tumor growth is glucose-depleted. Such conditions induce metabolic stress on T cells, which depends on glycolysis [8]. Further, accumulation of extracellular adenosine [9], accelerated potassium flux derived from necrosis of cancer cells [10], and tumor-released kynurenine are mediated by enzyme indoleamine 2.3-dioxygenase (IDO) as a byproduct of tryptophan metabolism, which all collectively impair effector T cell function and survival. In 2007, the CD4^+^ effector T cells were found to be important in antitumor response. The CD4^+^ T cells are able to eliminate tumors resistant to CD8^+^ T cell-mediated rejection, while the successful anticancer effect leading to tumor rejection is mediated by cooperation between CD8^+^ and CD4^+^ T cells [11,12]. Additionally, the CD4^+^ T cells were found to be key contributors that are important for the efficacy of anti-PD-L1/PD-1 immunotherapy [13]; therefore, a better understanding of the CD4^+^ T cell exhaustion mechanism may help to improve immunotherapy.

In this study, we show that CD4^+^ T cell exhaustion mediated by tumor cells results in a broad spectrum of transcriptomic changes. The high expression of *PD-1* and strong overexpression of *PD-L1* observed by us in these cells indicate the occurrence of the suicidal mechanism of T cell elimination by tumors. Furthermore, we find that the PD-L1 expression in CD4^+^ T cells exhausted by tumors may depend on the interplay between the SWI/SNF chromatin remodeling complex and polycomb-repressive complex 2 (PRC2). Additionally, as the angiogenesis-related genes such as *VEGF*s are found to be overexpressed in exhausted CD4^+^ T cells, our results clearly indicate that CD4^+^ T cells exhausted by tumor may be involved in neovascularization process of growing tumor. We also find that the cancer-dependent CD4^+^ T cell exhaustion process may be reversible. Collectively, our findings highlight the molecular and physiological events that occur during CD4^+^ T cell exhaustion, meaning they may serve as the basis for the improvement of cancer immunotherapy based on adoptive CAR-T transfer.

## 2. Materials and Methods

### 2.1. Cell Cultures

MDA-MB-231 and 786-O cell lines authenticated by Eurofins Company are in the possession of Department of the Experimental Immunotherapy Maria Sklodowska-Curie National Research Institute of Oncology (Warsaw, Poland). All lines are persistently monitored for mycoplasma contamination prior to experiments. MDA-MB-231 was cultured on DMEM Low Glucose w/Stable Glutamine w/Sodium Pyruvate medium (Biowest, Nuaillé, France) supplemented with 10% heat inactivated fetal bovine serum (Biowest) and 786-O was cultured on RPMI1640 medium (Biowest) supplemented with 10% heat-inactivated fetal bovine serum (Biowest).

### 2.2. CD4^+^ T Cells Isolation and Stimulation

CD4^+^ T cells were isolated from the whole blood of healthy, male donors not older than 36 y/o. Blood was obtained from the Regional Blood Center (Warsaw, Poland). Mononuclear blood cells were isolated from buffy coats by centrifugation on a Ficoll–Paque Plus (Amersham Biosciences, Piscataway, NJ, USA) density gradient and incubated for 35 min in plastic flasks to separate adherents and non-adherent cells. CD4^+^ T cells were isolated using a Dynabeads™ Regulatory CD4^+^/CD25^+^ Cell Kit (Invitrogen) and activated with a T Cell Activation/Expression Kit (Miltenyi Biotec, Bergisch Gladbach, Germany) according to the manufacturers’ instructions. Isolated CD4^+^ T cells were cultivated on AIM V™ medium (Gibco, Amarillo, TX, USA) supplemented with 10 U/mL interleukin-2 (R&D Systems) for 12 days. All cell cultures were maintained in a humidified incubator at 37 °C with 5% CO_2_.

### 2.3. Transcriptome Profiling (RNA Sequencing)

Total RNA samples from CD4^+^ T cells from two independent healthy donors were isolated using a ReliaPrep™ RNA Cell Miniprep System (Promega, Madison, WI, USA) including the DNAse digestion step according to the manual. RNA integrity was assessed with the Agilent RNA 6000 Nano Kit on a 2100 Bioanalyzer (Agilent, Santa Clara, CA, USA) followed by library preparation with the Ion AmpliSeq Transcriptome Human Gene Expression Panel (Thermo) according to the manufacturer’s protocol, as described previously [14]. The raw reads were processed using the Torrent Suite analysis pipeline and mapped to the hg19 human genome assembly. The reads corresponding to each gene were counted with htseq-count [15]. The data were normalized and the differential expression levels of various genes were determined by DESeq2 using default parameters according to the protocol used by Love et al., 2014 [16]. BAM file data from analysis are available in The European Nucleotide Archive repository under the accession number PRJEB41044.

### 2.4. Quantification of mRNA Expression

Total RNA was isolated as above. Reverse transcription of 1 μg of RNA into complementary DNA (cDNA) was performed using a Transcriptor First Strand Synthesis Kit (Roche) and oligo d(T) primer. The template was diluted 10 times for the qPCR reaction. The qPCR utilized iQ™ SYBR© Green Supermix (Bio-Rad) and standard protocol. Data were quantified using the ΔΔCt method [17]. Ubiquitin (UBC) was used as an internal control. All values are presented as means ± standard deviations (SD). The list of primers of analyzed genes is available in Supplementary Materials (Table S1). 

### 2.5. Co-Culture of Cancer Cells and CD4^+^ T-Cells

Co-culture of CD4^+^ T cells with cancer cells was performed in the media specific for each of the used cancer cell lines. Cancer cells were seeded around 70% confluency onto a T-75 cell culture bottle one day before the beginning of the co-culture (day 11). On day 12, CD4^+^ T cells were restimulated using a T Cell Activation/Expression Kit and 7.5 × 10^6^ cells were added to each culture bottle containing cancer cells or were cultured without cancer cells. Some of the CD4^+^ T cells were further cultured in the presence of IL-2 up to day 15 without restimulation.

After 72 h of co-culture with cancer cells, CD4^+^ T cells were gently suspended to be detached from cancer cells and collected (T15M). CD4^+^ T cells cultured w/o cancer cells were used as a control (T15). In selected experiments, CD4^+^ T cells separated from cancer cells on day 15 of culture were further cultured with cancer cells until day 18 (T18 + M) or without cancer cells until day 18 (T18-M).

Cells were counted three times before seeding and then harvested after culture.

### 2.6. Antibodies and Flow Cytometry

APC antihuman CD4, clone RPA-T4 was purchased from BioLegend. FITC antihuman CD3, clone SK7; PE antihuman CD8, clone SK1; PE antihuman CD279, clone MIH4; APC antihuman CD273, clone MIH18; and PE antihuman CD274, clone MIH1 were purchased from BD (Franklin Lakes, NJ, USA) To determine cell viability, cells were stained with 7-amino-actinomycin D (7-AAD, BD Pharmingen). Flow cytometry was performed using FACSCantoII or FACSLyric (both from BD (Franklin Lakes, NJ, USA). Data were analyzed using FACSDiva software (BD Biosciences).

### 2.7. Protein Analysis Using SDS-PAGE and Western Blot

Collected cells were counted at collection point and lysed in 1 × Leammli buffer. Samples were separated on SDS-PAGE gels and transferred onto PVDF membranes (0.45 μm pore dimension). Membranes were blocked in 5% non-fat dry milk (VWR) for 1 h at room temperature. Subsequently, membranes were incubated with primary antibodies overnight at 4 °C. The following primary antibodies were used according to the manufacturers’ instructions: anti-AMPK (#5832, Cell Signaling Technology), phosphorylated AMPK (*p*-AMPK) (#8208, Cell Signaling Technology), BAF155 (#11956, Cell Signaling Technology), BAF170 (#12760, Cell Signaling Technology), BRG1 (#3538, Cell Signaling Technology), BRM (#11966, Cell Signaling Technology), Ezh2 (#5246, Cell Signaling Technology), INI1 (#8545, Cell Signaling Technology), PD-1 (#86163, Cell Signaling Technology), PD-L1 (#13684, Cell Signaling Technology), SUZ12 (#3737, Cell Signaling Technology), IL-6 (#12153, Cell Signaling Technology), and IL-1β (#12703, Cell Signaling Technology). The membranes were washed three times with TBS-T (0.01%) and incubated with HRP-conjugated secondary antibody (antirabbit IgG, #1705046, Bio-Rad) for 1 h in room temperature. After washing three times with TBS-T, the signal was detected using a chemiluminescent substrate (Western Bright Quantum, Advansta) and visualized using a UVTech gel blot imaging system.

### 2.8. Chromatin Immunoprecipitation

At least 2 × 10^6^ cells were used for an antibody in each experiment. Collected cells were fixed using 1 mM BS_2_G and 1% formaldehyde. Cells were lysed in cell lysis buffer followed by nuclei lysis in 1×RIPA. Probes were sonicated 5 times for 10 min (30/30 s) and incubated with antibody overnight at 4 °C. Following antibodies were used: BRG1 (#3538, Cell Signaling Technology), BRM (#11966, Cell Signaling Technology), Ezh2 (#5246, Cell Signaling Technology), histone H3 (#ab180727, Abcam), and trimetylated histone H3 (#07-473 Millipore). The mixture of protein A and protein G magnetic beads (1:1) was added and incubated for 2 h at 4 °C. Beads were washed three times in wash buffer. DNA was eluted from the beads using high-salt (300 mM) elution buffer and incubated at 65 °C overnight. Subsequently, samples were treated with Proteinase K and DNA was precipitated using phenol–chloroform–isoamyl alcohol. The obtained DNA was used in the qPCR procedure using iQ™ SYBR Green Mastermix (Bio-Rad). The utilized primers are given in the Supplementary Materials (Table S1).

### 2.9. Proteome Identification from Medium Using Mass Spectrometry Method

To denature proteins, 300 µL of cell culture supernatant from T15 and T15M was added to 0.23 µg of urea for a final concentration of approximately 8 M. Samples were vortexed thoroughly before disulfide bridge reduction via 1 h incubation with 20 mM tris (2-carboxyethyl)phosphine (TCEP) at 37 °C. Proteins were transferred onto a Vivacon 10 kDa molecular weight cut-off filter (Sartorius Stedim Biotech, Goettingen, Germany) and digested according to a FASP protocol with minor modifications [18]. Samples were spun at 14,000× *g* for 30 min and washed with urea solution (8 M urea in 100 mM ammonium bicarbonate buffer) before cysteine blocking via 20 min incubation at room temperature with 50 mM s-methylmethanethiosulfonate. Proteins were washed three times with urea buffer and three times with 100 mM ammonium bicarbonate. After each addition, the samples were centrifuged for 30 min at 14,000× *g* until the cut-off filter was dry. Digestion was carried out overnight using 4 ug of trypsin/LysC mix (Promega GmbH, Mannheim, Germany) at 37 °C. Peptides were eluted from spin filters by two washes with 100 mM ammonium bicarbonate and one wash with 500 mM NaCl solution. The dried peptides were reconstituted in 60 µL of Evosep solvent A (0.1% formic acid in water) via 15 min sonication. 

Samples were analyzed using an LC-MS system composed of Evosep One (Evosep Biosystems, Odense, Denmark) directly coupled to an Orbitrap Exploris 480 mass spectrometer (Thermo Fisher Scientific, Bremen, Germany). Peptides were loaded onto disposable Evotips C18 trap columns (Evosep Biosystems, Odense, Denmark) according to the manufacturer’s protocol, with some modifications aimed to prevent the samples drying. Briefly, Evotips were activated with 25 µL of Evosep solvent B (0.1% formic acid in acetonitrile) via 1 min centrifugation at 600× *g* followed by 2 min incubation in 2-propanol. After equilibration with 25 µL of solvent A, 20 µL of each sample solution was loaded onto the solid phase. Bound peptides were washed with 50 µL and covered with 180 µL of solvent A. Chromatography was carried out at a flow rate 500 nL/min using the 44 min (30 samples per day) preformed gradient on an EV1106 analytical column (Dr Maisch C18 AQ, 1.9 µm beads, 150 µm ID, 15 cm long, Evosep Biosystems, Odense, Denmark). Data were acquired in positive mode with a data-dependent method using the following parameters. The MS1 resolution was set to 60,000 with a normalized AGC target of 300%, auto maximum inject time, and a scan range of 350 to 1400 m/z. For MS2, the resolution was set to 15,000 with a standard normalized AGC target, auto maximum inject time, and top 40 precursors within an isolation window of 1.6 m/z considered for MS/MS analysis. Dynamic exclusion was set at 20 s with an allowed mass tolerance of ±10 ppm, with a precursor intensity threshold of 5 × 10^3^. Precursors were fragmented in HCD mode with a normalized collision energy of 30%. The spray voltage was set to 2.1 kV, with a funnel RF level of 40 and heated capillary temperature of 275 °C.

All raw files were processed in Proteome Discoverer (PD) (version 2.4.0.305, Thermo Fisher Scientific, Bremen, Germany). Spectra were searched by Sequest HT against the Homo sapiens SwissProt database (version 2017-10-25, 42,252 sequences) and Proteome Discoverer contaminants database (version 2015_5, 298 sequences). The processing workflow included RC spectrum files, Minora Feature Detector, Spectrum Selector, Precursor Selector, Sequest HT, and Percolator. During the main search, methylthio (C) was set as a fixed modification, whereas oxidation (M), acetyl (N-term), and met-loss (M) were set as dynamic modifications. MS and MS/MS mass tolerances were set to 10 ppm and 0.02 Da, respectively. Trypsin digestion was set to full, with a maximum of two missed cleavages allowed. The target–decoy strategy was used to assess FDR, while the target FDR for Percolator was set to 0.01.

Consensus Workflow used MSF Files, Feature Mapper, Percursor Ions Quantifier, PSM Grouper, Peptide Validator, Peptide and Proteins Filter, Protein Annotation, Protein FDR Validator, Protein Scorer, Protein Grouping, Peptide in Protein Annotations, and Protein Marker programs. The peptide confidence filter was set to high and strict parsimony was used for protein grouping, with unique and razor peptides taking part in protein identification. The experiment was repeated twice with 2 independent biological replicates in 3 technical repeats. Data are available from the ProteomeXchange Consortium with accession number PXD026447.

### 2.10. Statistical Analysis

For statistical analysis, Kruskal–Wallis test, Chi-square, *t*-test, ANOVA, and Mann–Whitney tests were used to determine the normality distribution, differences between samples, and their significances.

## 3. Results

### 3.1. The CD4^+^ T Cells Inhibit Cancer Cell Growth but Concomitantly Cancer Cells Suppress Proliferation of the CD4^+^ T Cell Population

**Figure 1:**
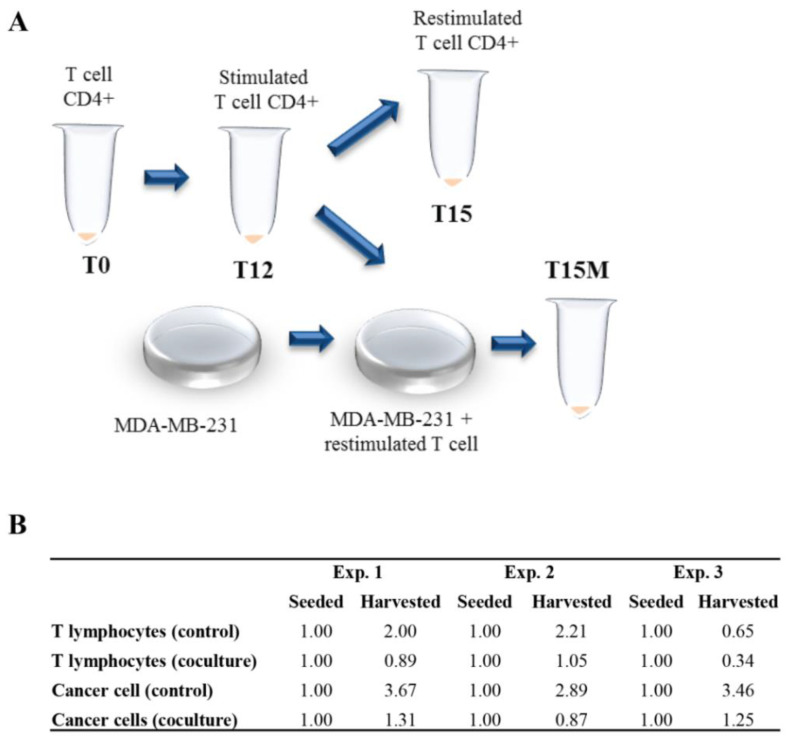
CD4^+^ T cells and MDA-MB-231 cancer cell growth is mutually inhibited in mixed cell cultures: (**A**) the schematic diagram of the experiment leading to the exhaustion of permanently activated CD4^+^ T cells, which is accelerated by MDA-MB-231 breast cancer cells; (**B**) data table showing 3 independent experiments performed with CD4^+^ T cells co-cultured with MDA-MB-231 breast cancer cells vs. CD4^+^ T cells cultured in the absence of cancer cells. The numbers of calculated cells are given in mln.

CD4^+^ T cells obtained from healthy donors were stimulated polyclonally with anti-CD3/CD28/CD2 moAb-coated beads in the presence of exogenous IL-2 for 12 days. CD4^+^ T cells collected after 12 days of culture (T12) were restimulated polyclonally without exogenous IL-2 for the next 3 days in the presence or without (T15M and T15, respectively) MDA-MB-231 breast cancer cell line expressing PD-L1 (Figure 1A and Figure S1). We discovered that in all experiments CD4^+^ T cells inhibited the cancer cell growth, resulting in about a three-fold reduction of cancer cell numbers compared to cancer cells growing without CD4^+^ T cells (Figure 1B). Additionally, the number of CD4^+^ T cells co-cultured with MDA-MB-231 cancer cells was dramatically reduced compared to CD4^+^ T cells growing in the absence of cancer (Figure 1B). In summary, the observed mutual growth inhibition of CD4^+^ T cells and MDA-MB-231 cancer cell line indicates the likely existence of the currently unrecognized mechanism of cancer-cell-dependent CD4^+^ T cell exhaustion. 

### 3.2. MDA-MB-231 Cancer Cells Affect the Global Transcriptome in CD4^+^ T Cells during Co-Culture

The CD4^+^ T cells obtained from two different healthy donors were polyclonally stimulated with anti-CD3/CD28/CD2 moAb-coated beads and with IL-2 for 12 days, then subsequently polyclonally restimulated for next 3 days without IL-2 in the presence or without MDA-MB-231 cancer cells. After 12 and 15 days of culture, cells were collected and RNA sequencing was performed. The detailed PCA analysis indicated global transcript changes, which were similar for both donors, for T12, T15, and T15M cell populations (Figure S2).

**Figure 2:**
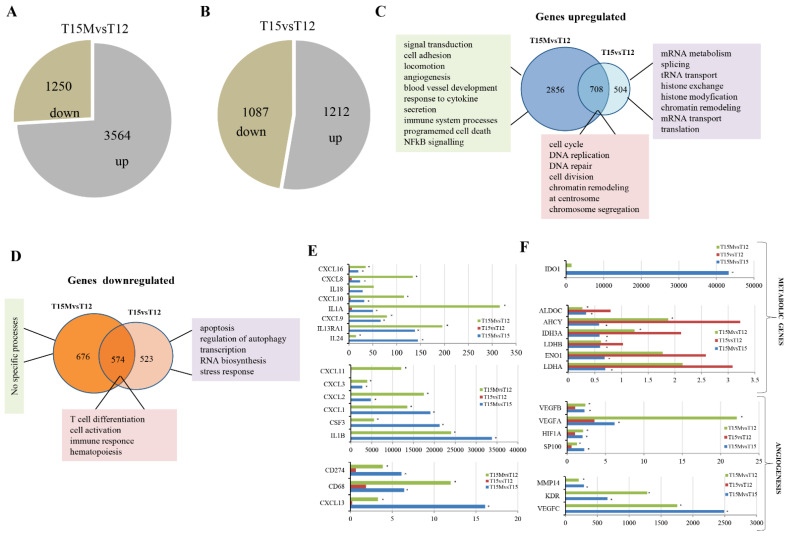
Cancer cells influence CD4^+^ T cell transcriptomes. The global transcriptomic analysis of differentially transcribed genes performed for (**A**) T15M versus T12 and (**B**) T15 versus T12. (**C**) Gene ontology analysis of genes upregulated in CD4^+^ T cells restimulated and grown with or without MDA-MB-231 breast cancer cells. (**D**) Gene ontology analysis of the genes downregulated in CD4^+^ T cells restimulated and grown with and without MDA-MB-231 breast cancer cells. (**E**) The expression of immune-related genes differentially expressed in CD4^+^ T cells for 12 days after stimulation and 3 days after restimulation with and without cancer cells. (**F**) The expression of metabolism and angiogenesis-related genes differentially expressed in CD4^+^ T cells on day 12 after stimulation and 3 days after restimulation with and without cancer cells. The fold changes are presented on x axis, while asterisks indicate statistical significance (*p* < 0.05).

The global transcriptomic analysis revealed that in CD4^+^ T cells co-cultured with MDA-MB-231 cancer cells (T15M), 3564 genes were upregulated and 1250 genes exhibited reduced expression levels when compared to CD4^+^ T cells stimulated for 12 days (T12) (Figure 2A). In contrast, the control CD4^+^ T cells stimulated for 12 days and restimulated for an additional 3 days without cancer cells (T15) exhibited less dramatic transcriptomic changes demonstrated by upregulation of 1212 and downregulation of 1097 genes when compared to CD4^+^ T cells stimulated for 12 days (T12) (Figure 2B), indicating that the presence of cancer cells has a stimulatory effect on CD4^+^ T cell transcriptomes.

The subsequent careful comparative analysis of transcriptomic alterations in T15M and T15 indicated that differentially expressed genes were specifically upregulated (2856) mostly in T15M compared to control T12 (Figure 2C). The Gene Ontology analysis indicated that the signal transduction, cell adhesion, locomotion, angiogenesis, blood vessel development, response to cytokine, secretion, immune system processes, programmed cell death, and NFkB-signaling-related genes were the most activated by co-cultivation with cancer cells (Figure 2C, Supplementary Materials, Table S2). In comparison, in T12 (restimulated CD4^+^ T cells growing without cancer cells), only 504 genes were specifically upregulated. These genes were involved mostly in processes such as mRNA metabolism, splicing, tRNA transport, histone exchange, histone modification, chromatin remodeling, mRNA transport, and translation (Supplementary Materials, Table S2). Surprisingly, the decreased expression of 676 genes was found in CD4^+^ T cells after co-culture with cancer cells (T15M) in comparison to 523 specifically downregulated genes in the control (T15). The 574 genes were commonly downregulated in both T15M and T15 (Figure 2D). Gene Ontology analysis revealed that only the genes downregulated in CD4^+^ T cells growing without cancer cells were defined in several processes such as apoptosis, regulation of autophagy, transcription, RNA biosynthesis, and stress response, while for T15M no defined processes were found (Figure 2D and Supplementary Materials, Table S2), further supporting the conclusion that co-cultivation of activated CD4^+^ T cells with cancer cells leads to specific upregulation of important regulatory processes, which may result in the cancer progression and immune system dysfunction.

This observation is further supported by the careful analysis of the class of immunomodulatory genes that are overexpressed in cells co-cultivated with T15M cancer cells when compared with control T15 and T12 CD4^+^ T cells (Figure 2E). Interestingly, the genes coding for chemokines such as *CXCL1*, *CXCL2, CXCL3,* and *CXCL8* (IL-8) were strongly upregulated in CD4^+^ T cells co-cultured with cancer cells only, further suggesting the direct influence of cancer cells on chemokine expression in CD4^+^ T cells. Interestingly, IL-8 overexpression may promote angiogenesis by recruiting the immunosuppressive cells to the tumor and stimulating the EMT process (epithelial-to-mesenchymal transition) [19]. On the other hand, the overexpression of the *CD274* gene encoding PD-L1 was found in deep transcriptome analysis, while the metabolism- and angiogenesis-related genes were upregulated in T15M samples compared to control T15 and T12 CD4^+^ cells, which were not exposed to cancer cells (Figure 2E,F). Among genes overexpressed in T15M CD4^+^ T cells, we found *IDO1* (Indoleamine 2,3-dioxygenase 1) to be the most upregulated gene. *IDO1* is overexpressed in various cancer types and is involved in suppression of effector T and NK cell function and in promotion of angiogenesis in solid tumors [20]. Additionally, the genes encoding glycolysis enzymes were found to be misregulated (mostly downregulated) in CD4^+^ T cells grown with cancer cells compared to controls. Intriguingly, almost all VEGFs were strongly overexpressed in T15M CD4^+^ T cells, suggesting that CD4^+^ T cells exhausted by cancer gain the ability to promote angiogenesis in solid tumor. The list of the most important cytokines and other immunomodulatory genes in T15M vs. T15 samples is shown in the Supplementary Materials (Table S3). The expression level of some cytokines with tumor promoting function was substantially elevated in T15M samples suggesting that cancer cells promote the expression of pro-inflammatory and pro-tumor factors. 

### 3.3. MDA-MB-231 Cancer Cells Promote Upregulation of Genes Encoding PD-1 and PD-1-Ligands in Persistently Activated CD4^+^ T Cells

**Figure 3:**
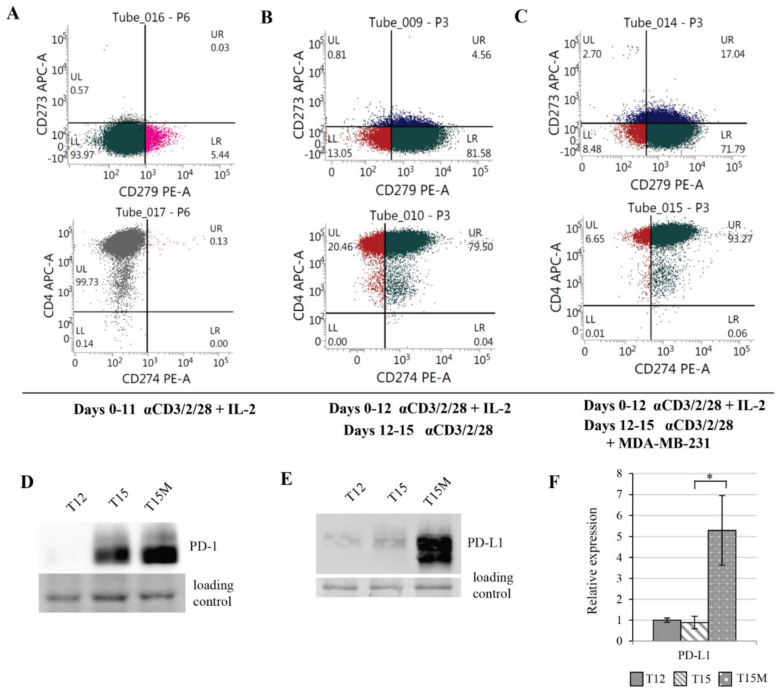
Prolonged polyclonal stimulation induced the expression of PD-1 (CD279), PD-L1 (CD274), and PD-L2 (CD273) in CD3^+^CD4^+^ T cells, which was additionally increased due their exposure to MDA-MB-231 cancer cells. Flow cytometry plots show the expression levels of CD279, CD273, CD274, and CD4 on the surfaces of (**A**) CD4^+^ T cells activated with anti-CD3/CD28/CD2 moAb-coated stimulatory beads and expanded in the presence of IL-2 (10 U/mL) for 11 days, (**B**) CD4^+^ T cells expanded with anti-CD3/CD28/CD2 Ab-coated stimulatory beads and with IL-2 (10 U/mL) for 12 days, restimulated on day 12 with anti-CD3/CD28/CD2 Abs without exogenous IL-2 and cultured up to day 15 in the absence of cancer cells, or (**C**) cultured up to day 15 in the presence of MDA-MB-231 cancer cells. (**D**) The protein levels of PD-1 and (**E**,**F**) PD-L1 were highly increased in CD4^+^ T cells when restimulation of lymphocytes was performed in the presence of MDA-MB-231 cancer cells. (**F**) The data from 3 independent biological replicates (donors) are presented, the statistical significance of which was calculated using Student’s *t*-test *p* < 0.05. Statistically significant values are indicated by asterisks. The uncropped blots and molecular weight markers are shown in Supplementary Materials.

Highly purified CD3^+^CD4^+^ T cells stimulated with anti-CD3/CD28/CD2 moAb-coated beads and expanded for 11 days with IL-2 (10 U/mL) consisted mostly of CD4^+^CD8^−^ T lymphocytes and a few double-positive CD4^+^CD8^+^ T cells (Figure S3). On day 11 of culture, the expanded CD4^+^ T cells did not express PD-L1 (CD274) or PD-L2 (CD273) on the cell surface, and only few lymphocytes expressed PD-1 (CD279) (Figure 3A and Figure S3). The further culturing of lymphocytes in the presence of exogenous IL-2 up to day 15 resulted in the induction of CD274, CD273, or CD279 expression in the expanded CD4^+^ T cells; however, still only a minority of cells expressed any of these markers (Figure S4). In contrast, the majority of lymphocytes that had been restimulated on day 12 with anti-CD3/CD28/CD2 moAb-coated stimulatory beads without exogenous IL-2 expressed CD279 or CD274 on the cell surface on day 15 (Figure S4). CD4^+^ T cells co-cultured with MDA-MB-231 cancer cells during the restimulation period exhibited substantially increased proportion of CD274^+^ and CD273^+^ T cells in comparison to lymphocytes restimulated in the absence of cancer cells (Figure 3B,C and Figure S4). The increases in the proportions of lymphocytes expressing CD279 on the cell surface were similarly high in cultures set with or without cancer cells (Figure 3B,C and Figure S4). In comparison to CD4^+^ T cells restimulated polyclonally in the absence of cancer cells, we found a profoundly greater increase in the production of PD-1 and PD-L1 proteins in CD4^+^ T cells restimulated in the presence of cancer cells (Figure 3D–F). A similar effect was found in CD4^+^ T cells restimulated and co-cultured with 786-O renal cancer cell line overexpressing PD-L1 on the cell surface (Figures S5 and S6), which strongly suggested the existence of universal mechanism of CD4^+^ T cell exhaustion induced by PD-L1-expressing cancers.

### 3.4. Exhaustion Process Induced by Cancer Cells Affects Expression of the SWI/SNF Chromatin Remodeling Complex, Glucose Metabolism, and Angiogenesis Genes

**Figure 4:**
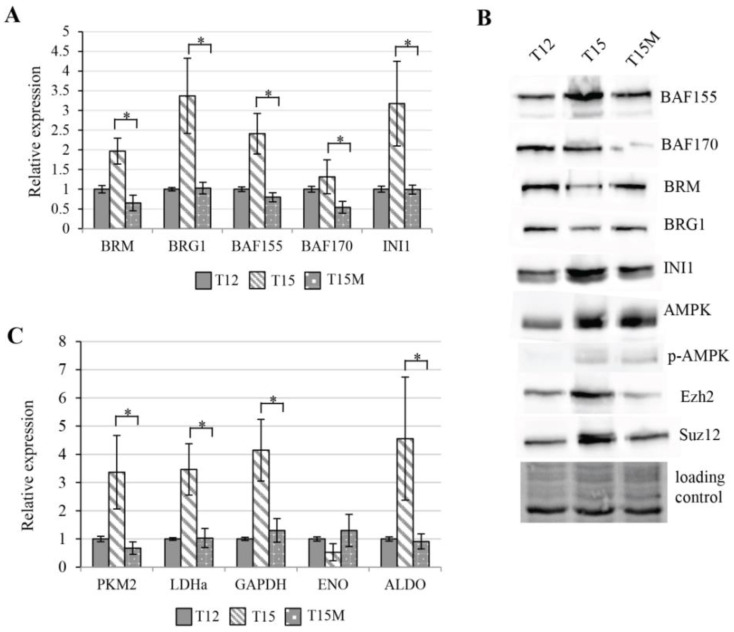
Cancer cells affect chromatin remodeling and glucose-metabolism-related genes in persistently activated CD4+ T cells: (**A**) expression levels of genes encoding SWI/SNF CRCs subunits in restimulated CD4^+^ T cells depend on the presence of cancer cells and are elevated in restimualted CD4^+^ T cells growing without cancer cells (T15); (**B**) protein abundance levels of SWI/SNF CRC and PRC2 core subunits, as well as key the metabolism controller AMPK, are affected in restimulated CD4^+^ T cells by the presence of cancer cells (T15M sample); (**C**) cancer cells affect the expression of metabolism related genes in restimulated CD4^+^ T cells (T15M) compared to restimulated CD4^+^ T cells (T15). (**A**,**C**) The data from 3 independent biological replicates (donors) are presented, the statistical significance of which was calculated using Student’s *t*-test *p* < 0.05, with the statistically significant values being indicated by asterisks. The uncropped blots and molecular weight markers are shown in Supplementary Materials.

The SWI/SNF chromatin remodeling complex (CRC) is known to be involved in transcription control via direct control of chromatin structure on promoter and other regulatory regions. As the global transcriptomic changes in CD4^+^ T cell were observed during exhaustion, the expression levels of genes encoding subunits of the SWI/SNF chromatin remodeling complex were measured. The elevated expression levels were found for genes encoding core subunits of SWI/SNF CRC in CD4^+^ T cells restimulated in the absence of cancer cells, while in CD4^+^ T cells restimulated in co-culture with cancer cells these genes were not activated, and indeed exhibited similar or even lower levels when compared to T12 CD4^+^ T cells. This observation suggests that the activation of CD4^+^ T cells requires enhanced chromatin remodeling, which is reduced during co-cultivation with cancer cells (Figure 4A). As the abundance of SWI/SNF subunits may be additionally regulated at the protein level, Western blot analysis was performed. The obtained results indicated that the protein levels of BRG1 and BRM ATPases were intact. while for BAF155, BAF170, and INI1 subunits of the SWI/SNF complex, the protein levels confirmed transcriptomic data, indicating aberrant function of SWI/SNF CRC during exhaustion in CD4^+^ T cells induced by MDA-MB-231 cancer cells (Figure 4B). Furthermore, since the PRC2 complex is known to be involved in T cell exhaustion and interplay with SWI/SNF, we checked the protein levels of the main PRC2 subunits. Interestingly, we found decreased levels of both Suz12 and EZH2 subunits in CD4^+^ T cells co-cultured with MDA-MB-231 cancer cells (Figure 4B). In chronically stimulated T cells, the PD-1 dependent signaling activates AMPK. Activated AMPK is involved in the mTOR inhibition. The mTOR pathway inhibition prevents T cells from the metabolic switching needed for their effector function and proliferation [21]. We found that restimulated CD4^+^ T cells growing either with or without cancer cells exhibited increased levels of AMPK and activated T172 P-AMPK (Figure 4B). It is known that the activation of T cells for rapid proliferation alters their metabolism, increases their glucose uptake, and accelerates glycolysis [22]; therefore, we examined the expression levels of certain genes encoding glycolysis enzymes such as PKM2, LDHA, ALDO, GAPDH, and ENO in CD4^+^ T cells stimulated for 12 days and subsequently restimulated for another 3 days and found increased expression of PKM2, LDHA, ALDO, and GAPDH. The expression of ENO was significantly decreased. Interestingly, the restimulated CD4^+^ T cells co-cultivated with MDA-MB-231 cells exhibited opposite regulation to T15. For PKM2, LDHa the expression levels observed in T15M were even lower than those observed for T12 (Figure 4C). Collectively, the observed alterations in machinery controlling the chromatin status, as well as the affected expression of glucose-metabolism-related genes, may serve as an attractive explanation for the reduced CD4^+^ T cell proliferation rate and suppressed effector function when CD4^+^ T cells were co-cultured with cancer cells.

**Figure 5:**
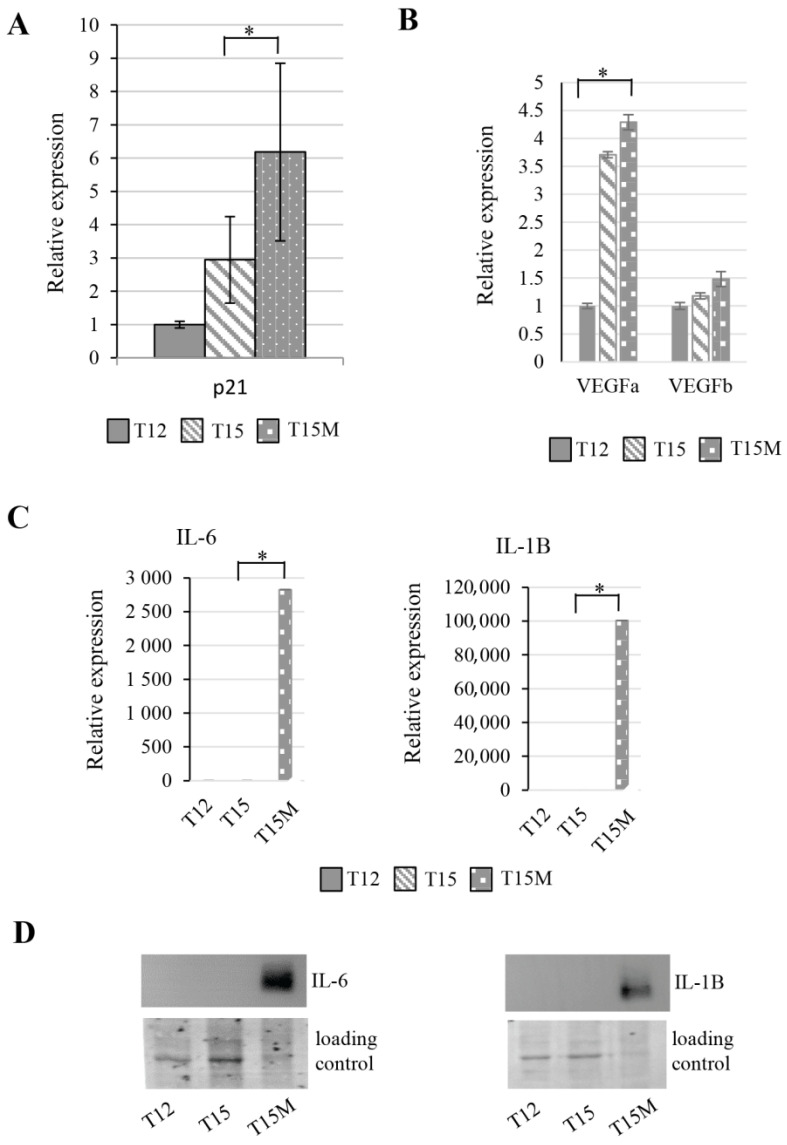
Exhausted CD4^+^ T cells revealed the increased expression of genes related to cell cycle, angiogenesis, and cytokines. During the process of exhaustion, restimulated CD4^+^ T cells exhibited altered expression of (**A**) cell cycle inhibitor p21, which was strongly elevated in CD4^+^ T cells cocultured with cancer cells, (**B**) upregulation of angiogenesis-related genes, and (**C**) overexpression of interleukins IL-6 and IL-1B only in CD4^+^ T cells co-cultured with cancer cells. (**D**) The highly elevated intracellular levels of IL-6 and IL-1B were found only in T cells co-cultured with cancer cells (T15M). (**A**) The data from 3 independent biological replicates (donors) are presented. (**B**,**C**) The data are from 1 representative biological replicate, the other biological replicates are presented in Figure S7. The statistical significance was calculated using Student’s *t*-test *p* < 0.05 and statistically significant values are indicated by asterisks. The uncropped blots and molecular weight markers are shown in Supplementary Materials.

The exhausted T cells lose their proliferation potential and their cell cycle is inhibited, which is demonstrated by the elevated expression level of p21, the cell cycle inhibitor. In restimulated CD4^+^ T cells, the p21 expression increases, although the co-culture of CD4^+^ T cells with MDA-MB-231 cancer cells induces a dramatic elevation of p21 (Figure 5A). This observation is in line with the reduction of CD4^+^ T cell population when grown with cancer cells. Similarly, the genes involved in angiogenesis were elevated in CD4^+^ T cells after restimulation and co-culture with cancer cells. The transcript measurements of VEGFa and VEGFb using RT-qPCR confirmed the transcriptomic data (Figure 5B). We found that the IL-6- and IL-1B-encoding genes were dramatically overexpressed in CD4^+^ T cells due to exhaustion induced by cancer cells (Figure 5C). Additionally, the intracellular IL-6 and IL-1B protein levels were also highly elevated in these cells (Figure 5D). Moreover, in mass spectrometry analysis performed on media collected from restimulated CD4^+^ T cells grown with cancer cells, the secreted cytokines and other factors such as IL-8, IL-6, CXCL1, and ICAM1 were found (Figure S8). These observations collectively indicate the strong de-regulation of CD4^+^ T cells by cancer cells.

### 3.5. The BRM Containing the SWI/SNF Chromatin Remodeling Complex Directly Controls the PD-L1 Locus in CD4^+^ T Cells

**Figure 6:**
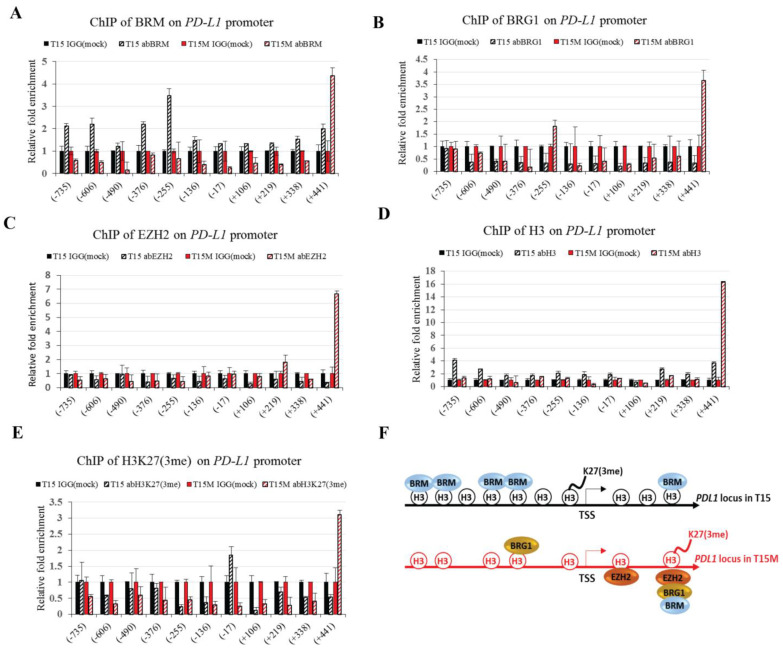
The chromatin status of the *PD-L1* locus varies upon CD4^+^ T cell exhaustion: (**A**) the presence of BRM ATPase of SWI/SNF CRCs on the *PD-L1* locus; (**B**) the presence of BRG1 ATPase of SWI/SNF CRCs on the *PD-L1* locus; (**C**) the presence of the EZH2 subunit of PRC2 on the *PD-L1* locus; (**D**) the presence of histone 3 (H3) on the *PD-L1* locus; (**E**) the presence of histone 3 lysine 27 trimethylation (H3K27me3) on the *PD-L1* locus; (**F**) model describing chromatin status on the *PD-L1* locus during exhaustion of CD4^+^ T cells. T15; CD4^+^ T cells restimulated for three days; T15M; CD4^+^ T cells restimulated in co-culture with cancer cells for 3 days. The experiment was repeated 3 times.

We found high overexpression of PD-L1 at both the transcriptomic and protein levels in CD4^+^ T cells co-cultured with MDA-MB-231 cancer cells. Consistently, the changes in epigenetic landscape were observed during T cell exhaustion; therefore, we decided to conduct the immunoprecipitation (ChIP) analysis for EZH2 and SWI/SNF subunits present on the *PD-L1* locus. Interestingly, the exchange of PRC2 subunit EZH2 and SWI/SNF ATPase subunits BRM and BRG1 was observed in CD4^+^ T cells restimulated and growing with cancer cells as compared to control without cancer cells. The binding of the BRM subunit was found in the promoter region of *PD-L1* only in restimulated CD4^+^ T cells, without co-cultivation with MDA-MB-231 cancer cells. The BRM target domains were located at −735, −606, −376, and −255 bp upstream of transcription start site (TSS); however, we found an additional target site for BRM in the *PD-L1* gene body at +441 bp downstream of TSS. Surprisingly, the BRM binding was not observed on the *PD-L1* promoter in CD4^+^ T cells co-cultured with cancer cells, with exception that we found enhanced recruitment of BRM +441 bp downstream of TSS (Figure 6A), although the binding at this position was labile and likely dependent on the exhaustion efficacy. Interestingly, the binding of the second SWI/SNF ATPase, BRG1, to PD-L1 was found only for CD4^+^ T cells co-cultured with cancer cells. The binding sites were located around −255 bp upstream of TSS and +441 downstream of TSS (Figure 6B). Interestingly, the co-cultivation of CD4^+^ T cells with cancer cells likely caused the exchange between BRM and BRG1-containing SWI/SNF CRC classes at position −255 bp. Additionally, EZH2, a main PRC2 subunit, was located +219 bp and +441 bp downstream from TSS on the *PD-L1* locus only for CD4^+^ T cells co-cultivated with cancer cells (Figure 6C). The binding +441 bp downstream of TSS suggested the parallel, likely cooperative binding of the BRG1-containing SWI/SNF class and PRC2 complex. Our results clearly indicated the repressive function of BRM in CD4^+^ T cells, which is replaced by the activatory role of the BRG1-containing SWI/SNF class during exhaustion. Moreover, the binding of SWI/SNF CRCs to the position +441 bp downstream on the *PD-L1* locus likely indicated the existence of a fine-tuning regulatory mechanism involving co-operation of BRM and BRG1-containing classes with PRC2 after stimulation by MDA-MB-231 cancer cells. We found differential histone 3 (H3) occupancy on the *PD-L1* locus. The H3 occupancy on the *PD-L1* promoter region was much more frequent in CD4^+^ T cells growing without cancer cells compared to CD4^+^ T cells co-cultured with cancer cells (Figure 6D). In contrast, the trimethylation of K27 in H3 (H3K27me3) was detected only at the −17 bp position upstream of TSS in CD4^+^ T cells co-cultivated with cancer cells, while in control cells the enrichment for H3K27me3 was found in the +441 bp position downstream of TSS (Figure 6E). Collectively, our findings strongly suggest the involvement of both PRC2 and SWI/SNF complexes in regulation of the *PD-L1* locus upon interaction with cancer cells. The higher H3 density in CD4^+^ T cells was consistent with low expression of *PD-L1* compared to CD4^+^ T cells exposed to cancer cells, which were characterized by decreased compaction of chromatin and shifted H3K27me3 (Figure 6F), highlighting the alteration of the chromatin status in CD4^+^ T cells upon exhaustion.

### 3.6. The CD4^+^ T Cell Exhaustion Process Is Reversible

**Figure 7:**
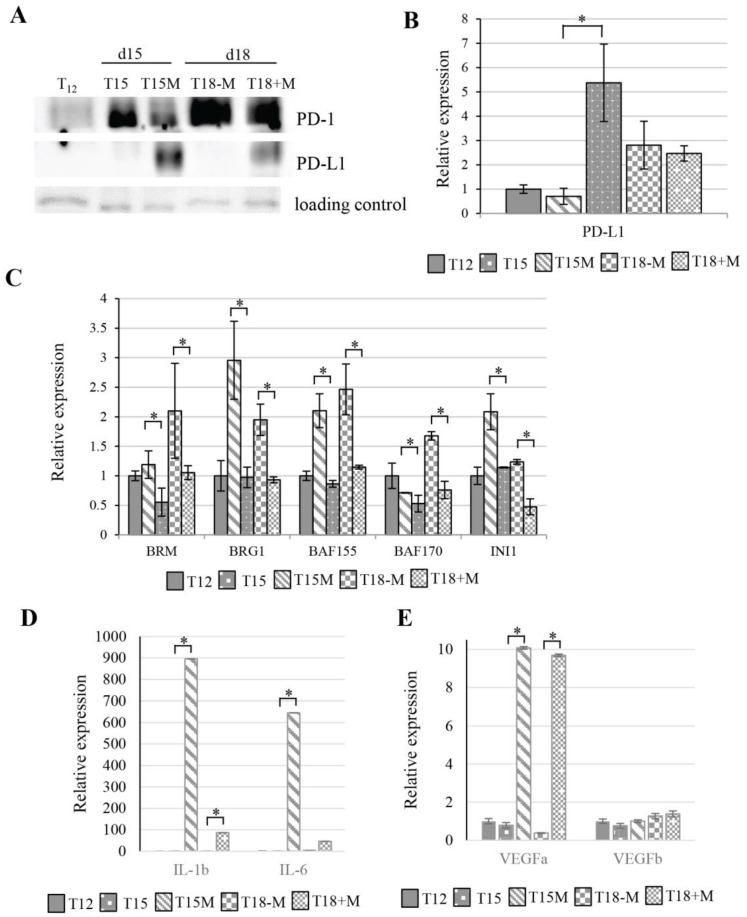
Cancer-cell-induced CD4^+^ T cell exhaustion may be reversed by removal of cancer cells. (**A**) PD-1 and PD-L1 protein levels and (**B**) PD-L1 expression in CD4^+^ T cells (T12) stimulated for 12 days after restimulation for 3 days with (T15M) and without (T15) cancer cells. Further T15M CD4^+^ T cells were removed from cancer cells and growing for next 3 days without (T18-M) and with (T18+M) cancer cells. (**C**) Expression of SWI/SNF subunits, (**D**) interleukins IL-1B and IL-6, and (**E**) angiogenesis-related encoding genes in T12, T15, T15M, T18-M, and T18+M CD4^+^ T cells. (**B**,**C**) The data from 3 independent biological replicates (donors) are presented. (**D**,**E**) The data are from 1 representative biological replicate. The statistical significance was calculated using Student’s *t*-test *p* < 0.05 and statistically significant values are indicated by asterisks. The uncropped blots and molecular weight markers are shown in Supplementary Materials.

The T cell exhaustion is a relatively long process that finally leads to T cell apoptosis or severe exhaustion, which was found in patients resistant to anti-PD-1/PD-L1 immune checkpoint therapy [23]. To estimate the exhaustion of CD4^+^ T cells in our model, CD4^+^ T cells restimulated for 3 days in the presence of cancer cells (T15M) were collected and split. Half of them were cultured with cancer cells for the next 3 days (T18+M) and half of them were cultured without cancer cells (T18-M). The restimulated CD4^+^ T cells growing with cancer from day 12 up to day 15 (T15M) exhibited PD-L1 overexpression at both the protein and transcript levels, although when these cells were separated from cancer cells on day 15 and further cultured without cancer cells (T18-M) the PD-L1 expression was inhibited. An opposite effect was found in T15M CD4^+^ T cells continuously cultured with cancer cells from day 15 to day 18 (T18+M). In the T18+M cell population, the PD-L1 expression was maintained at a high level (Figure 7A,B). These results suggest that the induction of PD-L1 expression in CD4^+^ T cells undergoing exhaustion depends on cancer cells, although the exact mechanism of the induction pathway remains unknown. The CD4^+^ T cells previously grown with cancer cells and separated from them for 3 days (T18-M) exhibited expression of most genes encoding core SWI/SNF complex subunits (*SMARCA2*/BRM, *SMARCA4*/BRG1, *SMARCC1*/BAF155, *SMARCC2*/BAF170) that were strongly elevated in comparison to T15M, whereas in T18+M the expression remained at the levels similar to those observed for T15M CD4^+^ T cells, indicating the strong effect on chromatin remodeling in CD4^+^ T cells upon release from the contact with cancer cells (Figure 7C). Similarly, the opposite regulatory effect was observed for genes encoding IL-1B, IL-6, and VEGFa in T18-M and T18+M CD4^+^ T cells. In contrast, VEGFb exhibited similar expression levels in all tested lines (Figure 7D,E). Collectively, our results indicated that transcriptional changes induced in CD4^+^ T cells by cancer cells may be reversible when the contact with cancer cells is discontinued. 

## 4. Discussion

**Figure 8:**
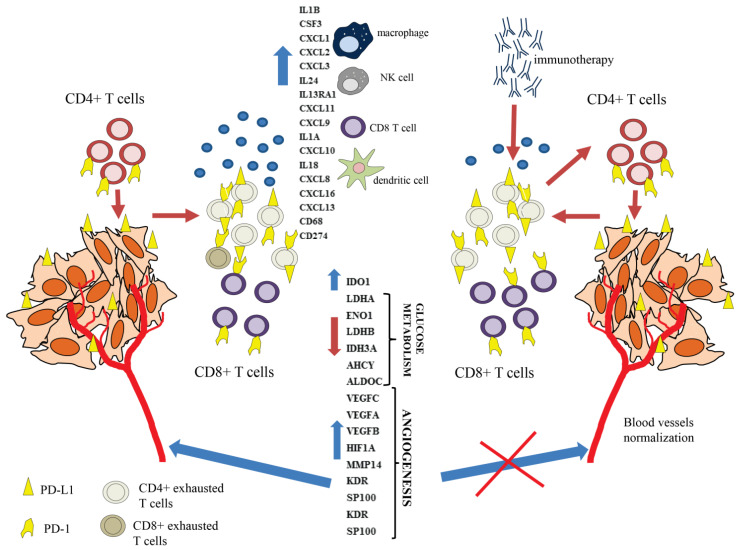
Based on our results, we postulate that persistently activated CD4^+^ T cells co-cultured with cancer cells become exhausted and overexpress various cytokine-encoding genes, which may facilitate recruitment of other immune cells such as the effector CD8^+^ T cells to the tumor microenvironment (TME). The CD4^+^ T cells during the exhaustion process express PD-L1 on their surfaces, which likely leads to elimination or exhaustion of effector CD8^+^ T cells. Additionally, exhausted CD4^+^ T cells may eliminate or exhaust other CD4^+^ T cells in TME and overexpress the proangiogenic factors. Immunotherapy activates exhausted CD4^+^ T cells, reducing the levels of proangiogenic factors and leading to blood vessels normalization.

A proper understanding of the mechanism behind T cell exhaustion is crucial not only for the establishment of more effective immunotherapy for treatment of various cancer types, but also for designing modern CAR-T therapy [24]. The autologous CAR-T therapy is currently used for treatment of hematological malignancies, although ‘off the shelf’ allogenic CAR-T therapy from healthy donors has many advantages and is developing extensively [25]. For proper cancer defense, both CD4^+^ T cells and CD8^+^ T cells are required [11,12]. CD8^+^ T cells become exhausted by cancer cells [26], although it was also demonstrated that CD4^+^ T cells also undergo exhaustion in cancer patients [27]. The phenomenon of CD8^+^ T cell exhaustion has been extensively investigated and some specific ‘exhaustion’ markers have been found, whereas the effector CD4^+^ T cell exhaustion process is still not well understood. In this paper, we present an attractive model of exhaustion of CD4^+^ T cells by PD-L1 expressing MDA-MB-231 and 786-O cancer cell lines. In our study, we found that the prolonged CD4^+^ T cell polyclonal stimulation leads to the induction of the exhaustion process, which is dramatically accelerated by cancer cells. The global transcriptomic analysis of restimulated CD4^+^ T cells grown with cancer cells compared to restimulated CD4^+^ T cells grown without cancer cells revealed that CD4^+^ T cells in contact with cancer cells overexpressed the chemokine and other cytokine-encoding genes, such as the *CXCL* family and interleukins IL-6, IL-1B, and IL-8, which may be secreted to the tumor microenvironment (TME). The increased cytokine level in TME may lead to the attraction of other cell populations of the immune system, e.g., CD8^+^ T cells, neutrophils, and macrophages, which consequently affects the TME composition, and depending on the dominant signals may create the immunosuppressive conditions enabling the tumor immune escape [28]. As was reported previously, many cytokines display both positive and negative roles in the anticancer immune response. These roles depend on the cellular context, differing receptor usage, and interference with other signals [29]. For example, IL-6 plays a dual function in TME. IL-6 may influence tumor cells through many downstream mediators, leading to support metastasis, survival, and proliferation of cancer cells. Within the tumor stroma, IL-6 may also act to promote tumor evasion and angiogenesis [30]. IL-1B has tumor-promoting effects in many cancer types via promotion and enhancement of angiogenesis and metastasis. A synergistic action of anti-PD-1 and anti-IL-1B therapy has been observed. This combined therapy leads to alterations of TME cell populations in favor of those with an antitumor effect [31]. During exhaustion of CD4^+^ T cells, high expression of the *CD274* gene encoding PD-L1 was found. The FACS and Western blot analyses revealed high PD-L1 expression on the cell surface, suggesting that exhausted CD4^+^ T cells may promote or accelerate the dysfunction, exhaustion, or elimination of still active CD4^+^ T cells, as well as active effector CD8^+^ T cells. CD4^+^ and CD8^+^ T cells may be eliminated by exhausted effector CD4^+^ T cells in TME by so-called suicide elimination. Additionally, the exhausted CD4^+^ effector T cells also overexpressed the *IDO1* (indoleamine 2,3-dioxygenase 1) gene involved in tryptophan metabolism. IDO1 is well known to be involved in metabolic T cell dysfunction or exhaustion mediated by kynurenine (a tryptophan metabolism intermediate produced by IDO1) [32]. Interestingly, in melanoma CD4^+^ T cells, exhaustion was found to be related to the kynurenine pathway and *IDO1* overexpression [33]. Interestingly, the overexpression of angiogenesis and development of blood-vessel-related genes, such as VEGFs, in restimulated CD4^+^ T cells co-cultured with cancer cells compared to control CD4^+^ T cells restimulated alone indicated the cancer-specific induction of these genes (Figure 8). The blockade of the immune checkpoint by immunotherapy caused activation of dysfunctional or exhausted T cells, leading to normalization of tumor blood vessels and tumor sensitization for other therapies such as radiotherapy, classical chemotherapy, and targeted therapy [34]. The mechanism of this phenomenon is still unrecognized, although in mouse models lacking CD4^+^ or CD8^+^ T cells the anti-CTLA-4 and anti-PD-1 treatment promoted normalization of tumor vessels via Th1 CD4^+^ helper T cell activation [35]. Moreover, in breast and colorectal cancer, it was found that the CD4^+^ T cells alone were insufficient for the induction of blood vessel remodeling, while the vascular normalization effect induced by immunotherapy was mediated by the activation of effector CD8^+^ T cells [36].

Epigenetic reprogramming is critical for CD8^+^ T cell exhaustion [37]. The ATP-dependent chromatin remodeling SWI/SNF complex (CRC) is responsible for gene expression control. We found that the expression of genes encoding the main subunits of SWI/SNF CRCs is decreased in exhausted CD4^+^ T cells. Interestingly, it is known that the SWI/SNF complex usually antagonizes the PRC2 complex, although for some genes it cooperates with the PRC2 complex, which is involved in T cell exhaustion. During the exhaustion process that occurred in CD4^+^ T cells grown with cancer cells, the PD-L1 expression was strongly elevated compared to control CD4^+^ T cells. The ChIP analysis revealed the changes in SWI/SNF complex occupancy and SWI/SNF class presence on the *PD-L1* locus. The BRM-containing class of SWI/SNF CRC and denser H3 occupancy were found on the *PD-L1* locus in the CD4^+^ T cells grown without cancer cells. In CD4^+^ T cells exhausted by cancer, instead of BRM-SWI/SNF CRC, the BRG1-containing class of SWI/SNF CRC was mainly detected on the *PD-L1* locus. Additionally, the H3 occupancy was reduced and the recruitment of the EZH2-containing PRC2 complex was found on *PD-L1*. The H3K27me3 position also changed from TSS to +441 bp downstream upon exhaustion. Collectively, our results suggest that an interplay between SWI/SNF CRCs and PRC2 is directly involved in *PD-L1* transcription control. For the *PD-L1* locus, the BRM-containing SWI/SNF CRC may act as a transcriptional repressor, while BRG1-containing SWI/SNF CRC and PRC2 may cooperate in the activation of PD-L1 expression during exhaustion. The exchange of SWI/SNF CRCs classes and their interplay with PRC2 likely results in changes in chromatin density (nucleosome occupancy) and the H3K23me3 position, although the signaling pathway or mechanism behind this exchange remains elusive. Here, we show that the CD4^+^ T cell exhaustion process induced by cancer cells may be reversible. If CD4^+^ T cells exhausted due to contact with cancer cells are separated from these cancer cells, they start to suppress the proangiogenic and cytokine-encoding genes and restore the expression of SWI/SNF CRC genes, resulting in the observed reduction of *PD-L1* expression. The understanding of the CD4^+^ T cell exhaustion process is of high importance not only for immunotherapy, but also in the context of CAR-T therapy, which might be applied for treatment of solid tumors. The involvement in epigenetic machineries such as SWI/SNF CRCs and PRC2 opens up the new possibility of modulation of the anticancer immune response via the use of epi-drugs such as EZH2 inhibitors. 

Comparison of persistently activated T cells undergoing exhaustion in the absence or in the presence of cancer cells suggested that juxtacrine signaling (contact-dependent signalling) from cancer cells initiates the process of T cell suppression directly; however, inhibitory signals delivered from cancer cells to T cells could induce changes in gene expression patterns in T cells, leading to production of inhibitory cytokines and enzymes that indirectly initiate paracrine suppression within T cell populations. We detected secreted cytokines in the medium of co-cultured T cells with cancer cells, suggesting a paracrine mechanism of T cell exhaustion. Given this observation, it is likely that the T cell exhaustion is caused by a combined mechanism involving both juxtacrine and paracrine signaling. This interpretation is supported by observation that functionality of T cells ‘rescued’ from tumor cells partially reverted to the state found in T cells not exposed to cancer cells.

## 5. Conclusions

In CD4^+^ T cell exhaustion induced by cancer cells, global transcriptomic changes occurred. The exhausted CD4^+^ T cells were characterized by upregulated expression of more than 3500 genes, while only 1250 genes were found to be downregulated. The upregulated genes were related to angiogenesis, immune system processes, locomotion, cell adhesion, and cytokines. Moreover, the secretion of some cytokines was observed in medium. The increased expression of PD-L1 was observed in exhausted CD4^+^ T cells, and its expression was dependent on contact with cancer cells. The expression of PD-L1 in exhausted CD4^+^ T cells suggests that they may eliminate or exhaust effector CD4^+^ and CD8^+^ T cells infiltrating tumors. The PD-L1 expression was found to be controlled by the interplay of chromatin remodeling SWI/SNF and PRC2 complexes. Moreover, we found that the exhaustion process is at least partially reversible when CD4^+^ T cells lose contact with cancer cells. This study opens up new important questions for further investigations, including whether cancer cells without PD-L1 expression can induce CD4^+^ T cell exhaustion in a similar or different way and whether the blockage of PD-1/PD-L1 interactions could reverse CD4^+^ T cell exhaustion.

## Data Availability

The NGS data are available from The European Nucleotide Archive repository under the accession number PRJEB41044; proteomic data are available from the ProteomeXchange Consortium with accession number PXD026447.

## Supplementary Materials

The following are available online at https://www.mdpi.com/article/10.3390/cancers13164148/s1, Figure S1: PD-L1 staining of MDA-MB-231 indicated its expression in this line, Figure S2: Principal component analysis for CD4+ T cell samples from two independent donors at day 12 after stimulation and at day 15 after 3 days of restimulation with and without cancer cells, Figure S3: CD3+CD4+ T cells activated with anti-CD3/CD28/CD2 Ab-coated beads and expanded with IL-2 (10 U/mL) for 11 days did not express CD274 and CD273 and only very few lymphocytes expressed CD279 on the cell surface, as assessed by flow cytometry analysis, Figure S4. MDA-MB-231 cancer cells promoted increased proportions of CD274+CD4+ T cells and CD273+CD4+ T cells but did not further enhance the proportion of CD279+CD4+ T cells during polyclonal restimulation of persistently activated CD4+ T cells, Figure S5: The restimulated CD4+ T cells isolated from healthy donors adhere to cancer cells in mixed cultures, Figure S6: The PD-L1 expression on restimulated CD4+ T cells is induced regardless of cancer cell type, Figure S7: The qRT-PCR analysis for IL-6, IL-1B, and VEGFs (a and b) in an additional two biological replicates. The statistical significance calculated using Student’s t-test at p < 0.05 is indicated by an asterisk, Figure S8: Co-cultivation of CD4+ T cells with cancer cells induce cytokines and secretion of other factors to the medium, Table S1: The list of primers used for qRT-PCR method and ChIP analysis, Table S2: Comparative analysis of transcriptomic changes. The GO analysis and list of genes upregulated in CD4+ T cells restimulated to day 15 and co-cultured with cancer cells vs. stimulated for 12 days—T15M vs. T12. The GO analysis and list of genes upregulated in CD4+ T cells restimulated to day 15 vs. those stimulated for 12 days—T15 vs. T12. The GO analysis and list of genes downregulated in CD4+ T cells restimulated to day 15 and co-cultured with cancer cells vs. stimulated for 12 days—T15M vs. T12. The GO analysis and list of genes downregulated in CD4+ T cells restimulated to day 15 vs. stimulated for 12 days—T15 vs. T12, Table S3: The list of the most important genes changed upon co-cultivation of CD4+ T cells with cancer cells (T15M) vs. CD4^+^ T cells without cancer cells (T15) after RNAseq analysis with *p* values and their function in cancer.
