# Supplementary material for: PD-L1 Overexpression, SWI/SNF Complex Deregulation, and Profound Transcriptomic Changes Characterize Cancer-Dependent Exhaustion of Persistently Activated CD4+ T Cells
Source: Cancers (Basel). 2021 Aug 18;13(16):4148. doi: 10.3390/cancers13164148 (PMC8391521; doi:10.3390/cancers13164148)
Supplement: Supplementary file 1 [file cancers-13-04148-s001.zip › Supplementary Table S1 and S3.pdf]

Table S1. The list of primers used for qRT-PCR method and ChIP analysis.

Method	Primer pair/*Gene*	Forward	Reverse
RT-qPCR	*ALDO*	5'-GACTCATCTGCAGCCAGGA-3'	5'-GAATTTCCTCTGAAGCACGC-3'
	BAF155	5'-ACTTGTAAGCAGCCCCAAGA-3'	5'-GCAGCTTCTTCAGTTCCAGG-3'
	BAF170	5'-ACAGACATCACCCGCTAGGT-3'	5'-ACGGCAAGAACAAGTCCAAG-3'
	BRG1	5'-GCAACAGTACTGCCAGCAAC-3'	5'-GACATTCCAGTCTCGACCCC-3'
	BRM	5'-TCCGAGGCAAAATCAGTCAAG-3'	5'-TTCCTCGATTTGGCCTTTTCT-3'
	ENO	5'-GCCTCCTGCTCAAAGTCAAC-3'	5'-AACGATGAGACACCATGACG-3'
	GAPDH	5'-GAAGGTGAAGGTCGGAGTC-3'	5'-GAAGATGGTGATGGGATTTC-3'
	IL-1b	5'-CTCCGGGACTCACAGCAAAA-3'	5'-TTTCAACACGCAGGACAGGT-3'
	IL-6	5'-TGCAATAACCACCCCTGACC-3'	5'-GTGCCATGCTACATTTGCC-3'
	INI1	5'-GACCAGGACAGGAACACGAG-3'	5'-CAAATGGAATGTGTGCCGG-3'
	LDHa	5'-GCCAGAGACAATCTTTGGTG-3'	5'-GGCCTGTGCCATCAGTATCT-3'
	p21 (CDKN1A)	5'-AAGACCATGTGGACCTGTCA-3'	5'-GGCTTCCTCTTGGAGAAGAT-3'
	PD-L1	5'-AAATGGAACCTGGCGAAAGC-3'	5'-GATGAGCCCCTCAGGCATTT-3'
	PKM2	5'-GTCTGAATGAAGGCAGTCCC-3'	5'-TGCAGTGGAGCTCAGAGAGA-3'
	UBC	5'-ATTTGGGTCGCGGTTCTTG-3'	5'-TGCCTTGACATTCTCGATGGT-3'
	VEGFa	5'-AGTACCCTGATGAGATCGAG-3'	5'-CTTTCTTTGGTCTGCATTCAC-3'
	VEGFb	5'-GAGATGTCCCTGGAAGAACACA-3'	5'-GAGTGGGATGGGTGATGTCAG-3'
ChIP-qPCR	Satach4	5'-CTGCACTACCTGAAGAGGAC-3'	5'-GATGGTTCAACACTCTTACA-3'
	CD274_1	5'-CTAGAAGTTCAGCGCGGGAT-3'	5'-GGCTGCGGAAGCCTATTCTA-3'
	CD274_2	5'-TTCCGCAGCCTTAATCCTTA-3'	5'-CGTGGATTCTGTGACTTCCTC-3'
	CD274_3	5'-CGAGGAACTTTGAGGAAGTCA-	5'-

		3’	AAAGTCAGCAGCAGACCCATA-3’
	CD274_4	5’-ATGGGTCTGCTGCTGACTTT-3’	5’-ACCTTCAGAGGGGTAAGAGC-3’
	CD274_5	5’-ACCCCTCTGAAGGTAAAATCAA-3’	5’-ACCCCTCTGAAGGTAAAATCAA-3’
	CD274_6	5’-TTCCCGGTGAAAATCTCATT-3’	5’-TCCTGACCTTCGGTGAAATC-3’
	CD274_7	5’-CGAAGGTCAGGAAAGTCCAA-3’	5’-GAAGCTGCGCAGAACTGG-3’
	CD274_8	5’-CAGTTCTGCGCAGCTTCC-3’	5’-GACCCTCCGTCCTAAAGTGC-3’
	CD274_9	5’-TAGCTCGCTGGGCACTTTAG-3’	5’-CAGATGCATTGCCTGTTCTT-3’
	CD274_10	5’-AATGCATCTGGCCTTCCTC-3’	5’-TGAACACACAGTCGCATAAGAA-3’
	CD274_11	5’-TGCGACTGTGTGTTCAGAAT-3’	5’-ATGCTGCAGCTGGGATAACT-3’

Table S3. The list of most important genes changed uppon co-cultivation of CD4+ T cells with cancer cells (T15M) vs CD4+ T cells without cancer cells (T15).

Gene name	Fold change (T15MvsT15)	pValue	Function in cancer	References
IL1A	50.11281	6.87E-05	Inflammatory factor, pro-tumor and anti-tumor effect	Baker KJ, Houston A and Brint E (2019) IL-1 Family Members in Cancer; Two Sides to Every Story. Front. Immunol. 10:1197. doi: 10.3389/fimmu.2019.01197.
IL1B	33799.64	0.001665	Pro-inflammatory, pro-tumorigenic and anti-tumor function depending on cell type and tumor stage	Pereira, J.F.S.; Jordan, P.; Matos, P. A Signaling View into the Inflammatory Tumor Microenvironment. Immuno 2021, 1, 91-118. https://doi.org/10.3390/immuno1020007, Jang, JH., Kim, DH. & Surh, YJ. Dynamic roles of inflammasomes in inflammatory tumor microenvironment. npj Precis. Onc. 5, 18 (2021). https://doi.org/10.1038/s41698-021-00154-7.
IL6	3916.794	4.99E-20	Pro-inflammatory role, tumor promoting function and anti-tumor function	Toshio Hirano, IL-6 in inflammation, autoimmunity and cancer, International Immunology, Volume 33, Issue 3, March 2021, Pages 127–148, https://doi.org/10.1093/intimm/dxaa078 Fisher, D.T.; Appenheimer, M.M.; Evans, S.S. The two faces of IL-6 in the tumor microenvironment. Semin. Immunol. 2014, 26, 38–47, doi:10.1016/j.smim.2014.01.008.

IL9	0.216019	0.056806	Pro-tumor and anti-tumor effect	Wan J, Wu Y, Ji X, Huang L, Cai W, Su Z, Wang S, Xu H. IL-9 and IL-9-producing cells in tumor immunity. Cell Commun Signal. 2020 Mar 30;18(1):50. doi: 10.1186/s12964-020-00538-5. PMID: 32228589; PMCID: PMC7104514.
IL15	3.093425	0.014264	Tumor promoting function	Fiore PF, Di Matteo S, Tumino N, et all. Interleukin-15 and cancer: some solved and many unsolved questionsJournal for ImmunoTherapy of Cancer 2020;8:e001428. doi: 10.1136/jitc-2020-001428
IL24	144.3807	1.14E-18	Anti-tumor function	Ma YF, Ren Y, Wu CJ, Zhao XH, Xu H, Wu DZ, Xu J, Zhang XL, Ji Y. Interleukin (IL)-24 transforms the tumor microenvironment and induces anticancer immunity in a murine model of colon cancer. Mol Immunol. 2016 Jul;75:11-20. doi: 10.1016/j.molimm.2016.05.010. Epub 2016 May 19. PMID: 27209087.
CCL20	172.3063	1.87E-18	Tumor promoting function	Kadomoto S, Izumi K, Mizokami A. The CCL20-CCR6 Axis in Cancer Progression. Int J Mol Sci. 2020 Jul 22;21(15):5186. doi: 10.3390/ijms21155186. PMID: 32707869; PMCID: PMC7432448.
CXCL1	19045.09	0.002966	Tumor promoting function	Yu S, Yi M, Xu L, Qin S, Li A and Wu K (2020) CXCL1 as an Unfavorable Prognosis Factor Negatively Regulated by DACH1 in Non-small Cell Lung Cancer. Front. Oncol. 9:1515. doi: 10.3389/fonc.2019.01515, Wang, N., Liu, W., Zheng, Y. et al. CXCL1 derived from tumor-associated macrophages promotes breast cancer metastasis via activating NF-κB/SOX4 signaling. Cell Death Dis 9, 880 (2018). https://doi.org/10.1038/s41419-018-0876-3
CXCL2	4821.445	1.61E-13	Tumor promoting function	Feliciano, P. CXCL1 and CXCL2 link metastasis and chemoresistance. Nat Genet 44, 840 (2012). https://doi.org/10.1038/ng.2379, Zhang F, Jiang J, Xu B, Xu Y, Wu C. Over-expression of CXCL2 is associated with poor prognosis in patients with ovarian cancer. Medicine. 2021;100:4(e24125).
CXCL3	2755.866	6.34E-25	Tumor promoting function	Gui SL, Teng LC, Wang SQ, Liu S, Lin YL, Zhao XL, Liu L, Sui HY, Yang Y, Liang LC, Wang ML, Li XY, Cao Y, Li FY, Wang WQ. Overexpression of CXCL3 can enhance the oncogenic potential of prostate cancer. Int Urol Nephrol. 2016 May;48(5):701-9. doi: 10.1007/s11255-016-1222-2. Epub 2016 Feb 2. PMID: 26837773, Qi YL, Li Y, Man XX, Sui HY, Zhao XL, Zhang PX, Qu XS, Zhang H, Wang BX, Li J, Qi SF, Jia LL, Luan HY, Zhang CB, Wang WQ. CXCL3 overexpression promotes the tumorigenic potential of uterine cervical cancer cells via the MAPK/ERK pathway. J

				Cell Physiol. 2020 May;235(5):4756-4765. doi: 10.1002/jcp.29353. Epub 2019 Oct 30. PMID: 31667838.
CXCL5	577.3176	0.055265	Tumor promoting function	Zhang W, Wang H, Sun M, et al. CXCL5/CXCR2 axis in tumor microenvironment as potential diagnostic biomarker and therapeutic target. Cancer Communications. 2020;40:69–80. 10.1002/cac2.12010
CXCL8	23.43699	5.16E-06	Pro-inflammatory, induces proliferation and prevent apoptosis of cancer cells	Pereira, J.F.S.; Jordan, P.; Matos, P. A Signaling View into the Inflammatory Tumor Microenvironment. Immuno 2021, 1, 91-118. https://doi.org/10.3390/immuno1020007.
CXCL9	66.22369	1.89E-06	Pro-tumor and anti-tumor effect	Neo SY, Lundqvist A. The Multifaceted Roles of CXCL9 Within the Tumor Microenvironment. Adv Exp Med Biol. 2020;1231:45-51. doi: 10.1007/978-3-030-36667-4_5. PMID: 32060845.
CXCL10	31.79693	8.02E-22	Pro-tumor and anti-tumor effect	Tokunaga R, Zhang W, Naseem M, Puccini A, Berger MD, Soni S, McSkane M, Baba H, Lenz HJ. CXCL9, CXCL10, CXCL11/CXCR3 axis for immune activation - A target for novel cancer therapy. Cancer Treat Rev. 2018 Feb;63:40-47. doi: 10.1016/j.ctrv.2017.11.007. Epub 2017 Nov 26. PMID: 29207310; PMCID: PMC5801162.
CXCL11	101.7608	0.000101	Pro-tumor and anti-tumor effect	Tokunaga R, Zhang W, Naseem M, Puccini A, Berger MD, Soni S, McSkane M, Baba H, Lenz HJ. CXCL9, CXCL10, CXCL11/CXCR3 axis for immune activation - A target for novel cancer therapy. Cancer Treat Rev. 2018 Feb;63:40-47. doi: 10.1016/j.ctrv.2017.11.007. Epub 2017 Nov 26. PMID: 29207310, Cao Y, Jiao N, Sun T, Ma Y, Zhang X, Chen H, Hong J and Zhang Y (2021) CXCL11 Correlates With Antitumor Immunity and an Improved Prognosis in Colon Cancer. Front. Cell Dev. Biol. 9:646252. doi: 10.3389/fcell.2021.646252.
CXCL13	16.07915	3.5E-09	Pro-tumor and anti-tumor effect	Hussain M, Adah D, Tariq M, Lu Y, Zhang J, Liu J. CXCL13/CXCR5 signaling axis in cancer. Life Sci. 2019 Jun 15;227:175-186. doi: 10.1016/j.lfs.2019.04.053.
CXCL16	19.54248	2.22E-06	Pro-tumor and anti-tumor effect	Korbecki J, Bajdak-Rusinek K, Kupnicka P, Kapczuk P, Simińska D, Chlubek D, Baranowska-Bosiacka I. The Role of CXCL16 in the Pathogenesis of Cancer and Other Diseases. Int J Mol Sci. 2021 Mar 28;22(7):3490. doi: 10.3390/ijms22073490.
CD274	6.094112	1.48E-07	Promotes tumor immune evasion	Han Y, Liu D, Li L. PD-1/PD-L1 pathway: current researches in cancer. Am J Cancer Res. 2020 Mar 1;10(3):727-742.
CTLA4	2.771362	0.032166	Promotes tumor immune evasion	Liu Y, Zheng P. How Does an Anti-CTLA-4 Antibody Promote Cancer Immunity? Trends Immunol. 2018 Dec;39(12):953-956. doi:

				10.1016/j.it.2018.10.009.
VEGFA	6.185286	4.03E-10	Induces angiogenesis	Apte RS, Chen DS, Ferrara N. VEGF in Signaling and Disease: Beyond Discovery and Development. Cell. 2019 Mar 7;176(6):1248-1264. doi: 10.1016/j.cell.2019.01.021.
VEGFB	2.258515	0.000455	Induces angiogenesis	Apte RS, Chen DS, Ferrara N. VEGF in Signaling and Disease: Beyond Discovery and Development. Cell. 2019 Mar 7;176(6):1248-1264. doi: 10.1016/j.cell.2019.01.021.
VEGFC	2489.806	1.65E-14	Induces angiogenesis	Apte RS, Chen DS, Ferrara N. VEGF in Signaling and Disease: Beyond Discovery and Development. Cell. 2019 Mar 7;176(6):1248-1264. doi: 10.1016/j.cell.2019.01.021.
IDO1	43270.02	8.01E-25	Promotes tumor immune evasion	Yao Y, Liang H, Fang X, Zhang S, Xing Z, Shi L, Kuang C, Seliger B, Yang Q. What is the prospect of indoleamine 2,3-dioxygenase 1 inhibition in cancer? Extrapolation from the past. J Exp Clin Cancer Res. 2021 Feb 8;40(1):60. doi: 10.1186/s13046-021-01847-4.
MMP1	22316.05	8.83E-23	Promotion of tumor progression	Quintero-Fabián S, Arreola R, Becerril-Villanueva E, Torres-Romero JC, Arana-Argáez V, Lara-Riegos J, Ramírez-Camacho MA and Alvarez-Sánchez ME (2019) Role of Matrix Metalloproteinases in Angiogenesis and Cancer. Front. Oncol. 9:1370. doi: 10.3389/fonc.2019.01370
CSF3	21270.7	1.91E-21	Tumor promoting effect	Saunders AS, Bender DE, Ray AL, Wu X, Morris KT (2021) Colony-stimulating factor 3 signaling in colon and rectal cancers: Immune response and CMS classification in TCGA data. PLOS ONE 16(2): e0247233. https://doi.org/10.1371/journal.pone.0247233
