# Supplementary figures and images for: PD-L1 Overexpression, SWI/SNF Complex Deregulation, and Profound Transcriptomic Changes Characterize Cancer-Dependent Exhaustion of Persistently Activated CD4+ T Cells
Source: Cancers (Basel). 2021 Aug 18;13(16):4148. doi: 10.3390/cancers13164148 (PMC8391521; doi:10.3390/cancers13164148)

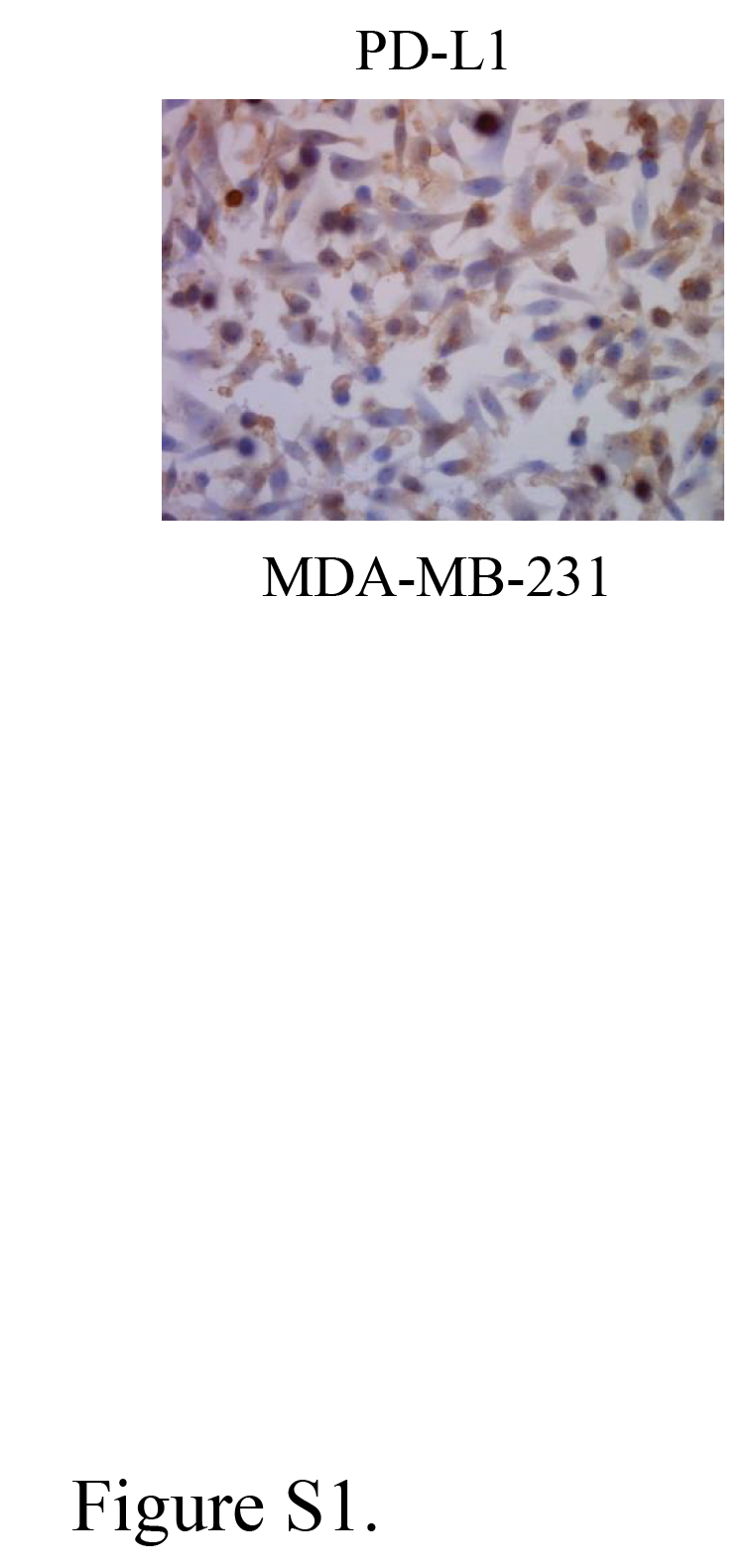

Supplement: Supplementary file 1 [file cancers-13-04148-s001.zip › Fig S1.tif]

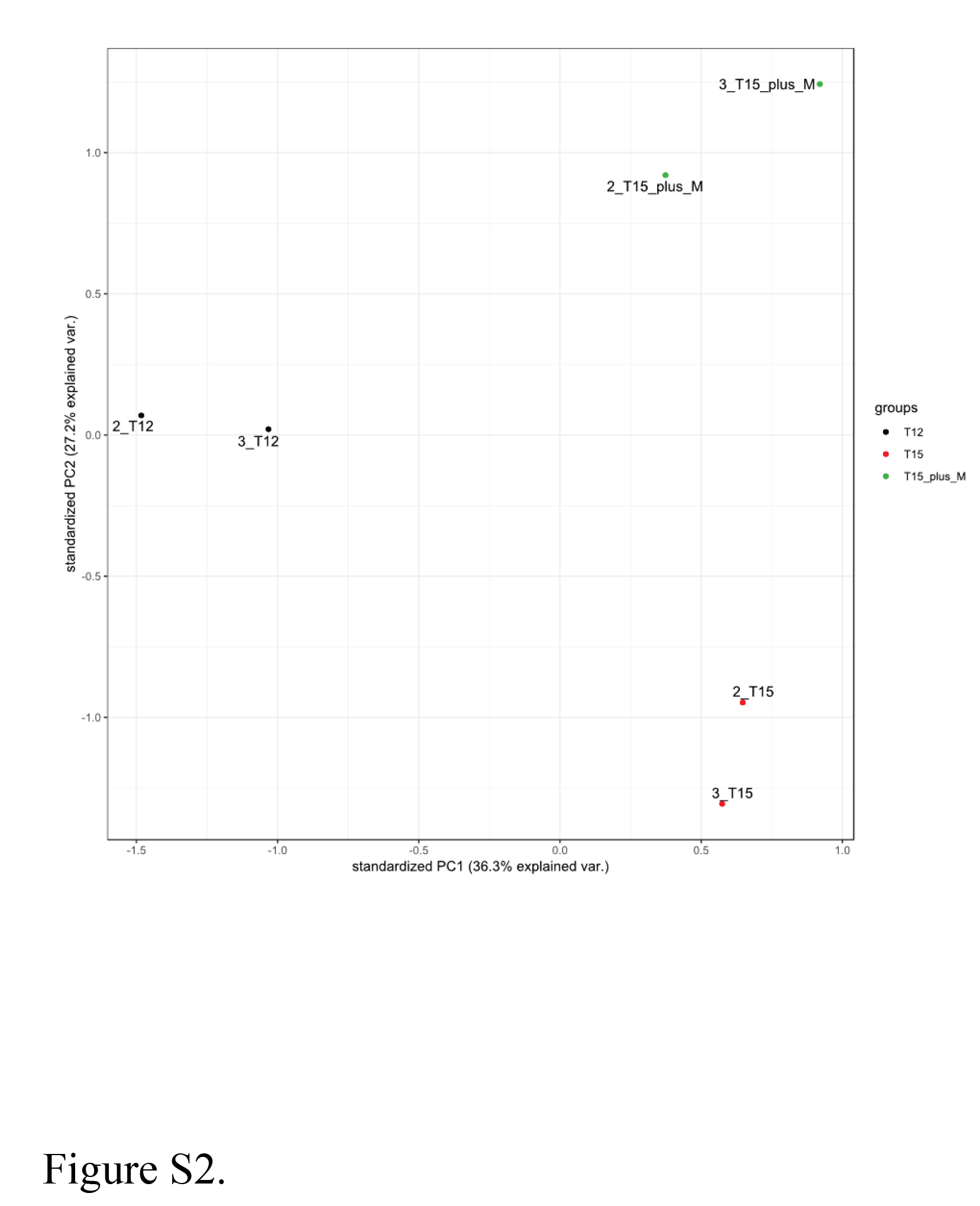

Supplement: Supplementary file 1 [file cancers-13-04148-s001.zip › Fig S2.tif]

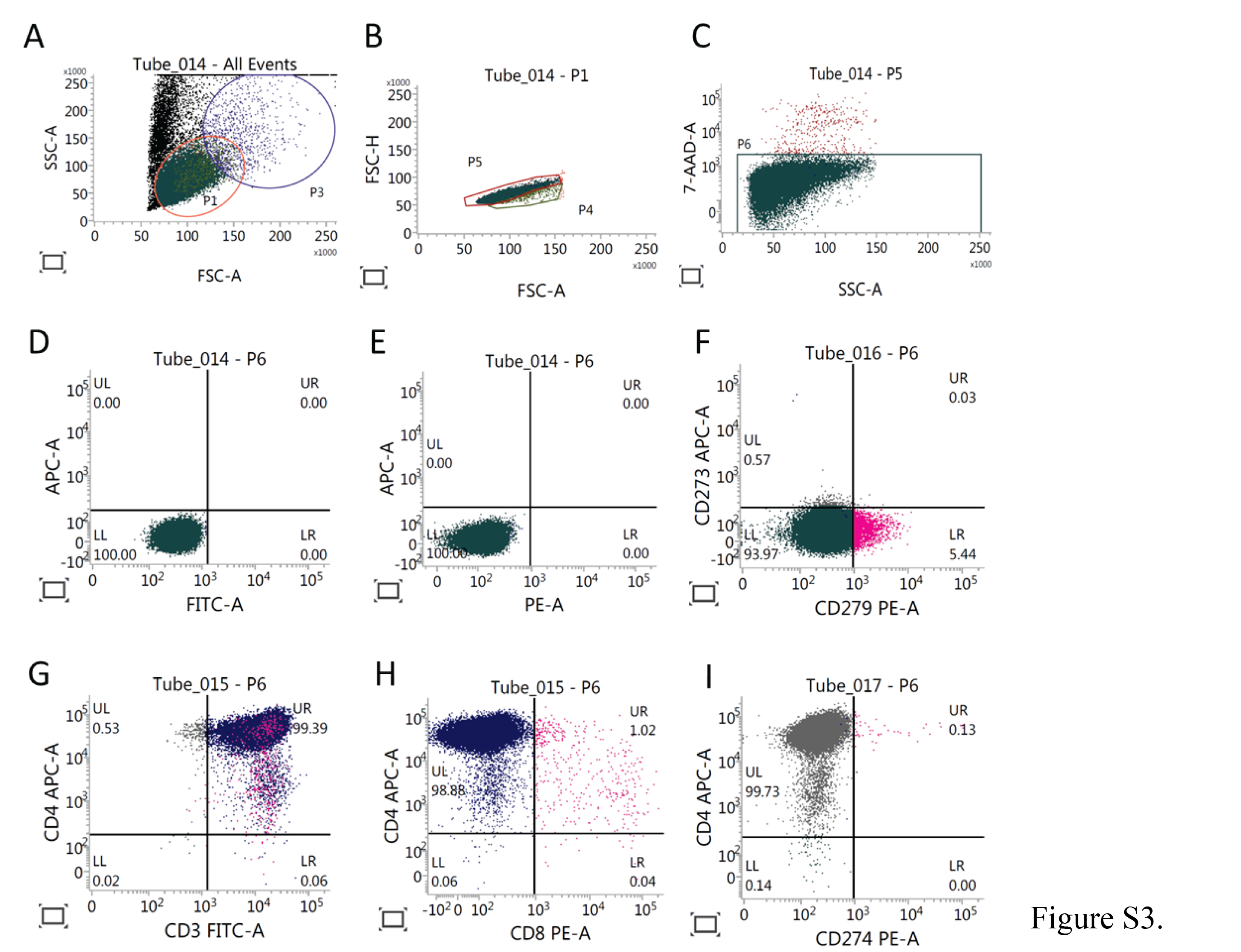

Supplement: Supplementary file 1 [file cancers-13-04148-s001.zip › Fig S3.tif]

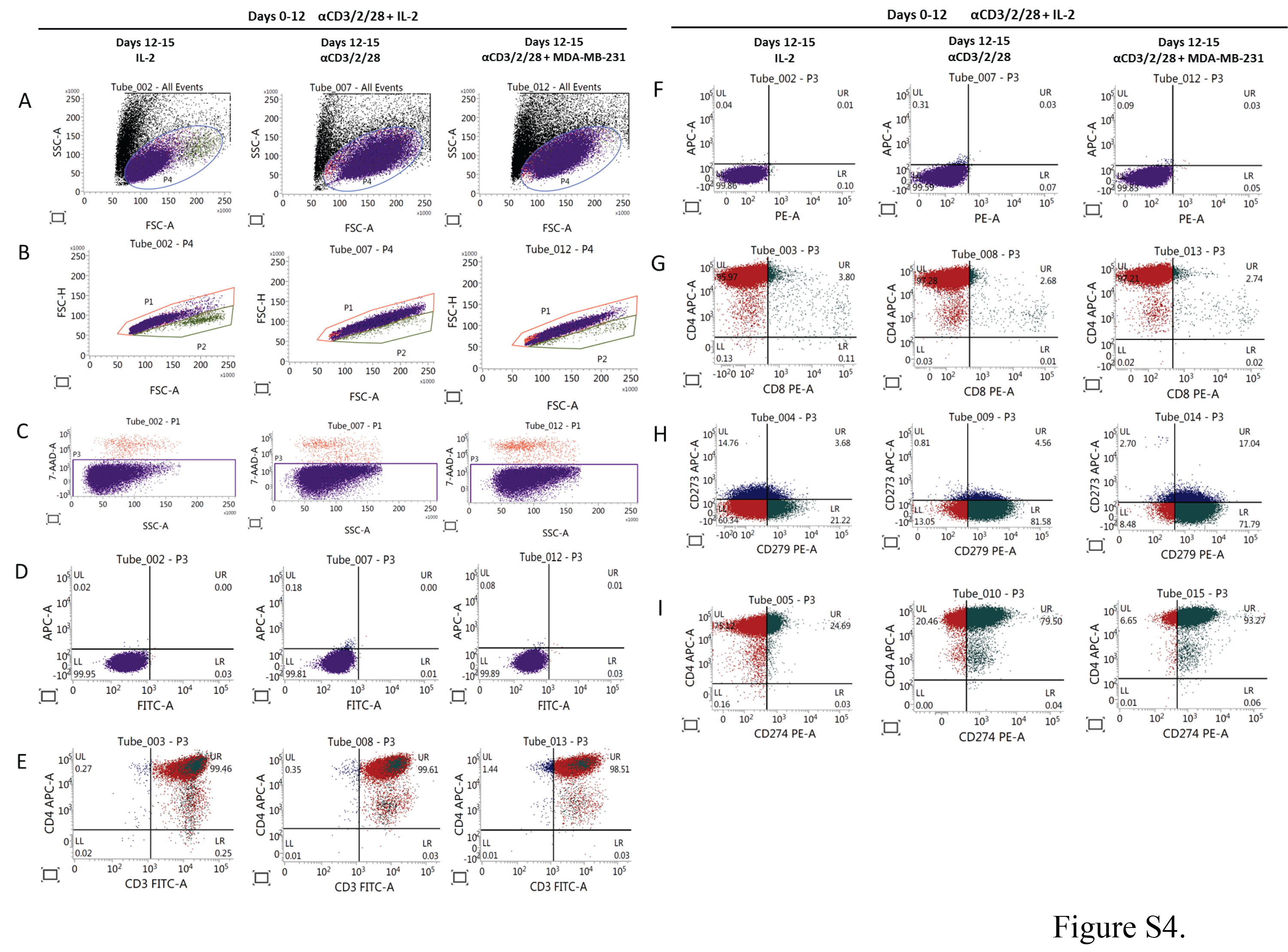

Supplement: Supplementary file 1 [file cancers-13-04148-s001.zip › Fig S4.tif]

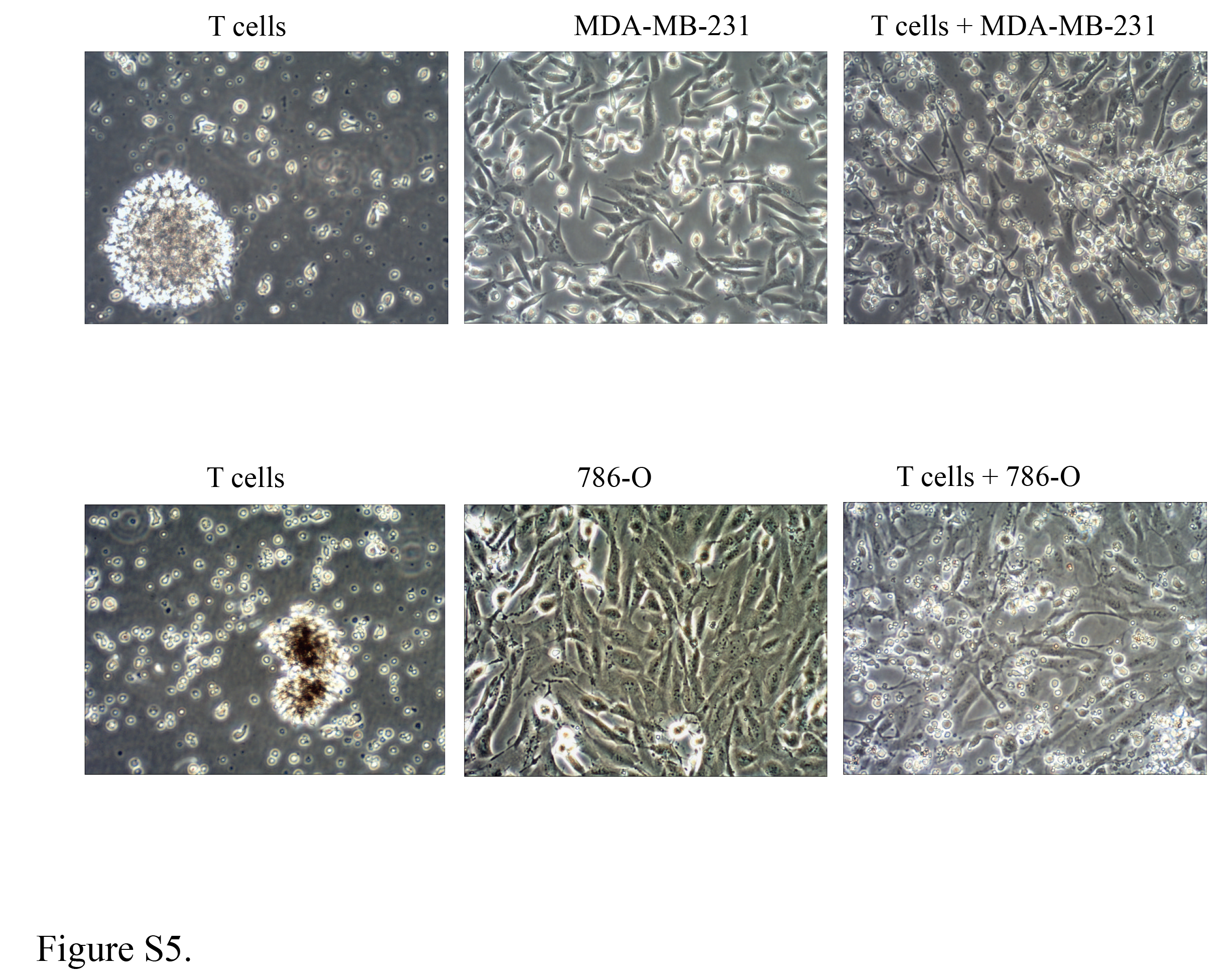

Supplement: Supplementary file 1 [file cancers-13-04148-s001.zip › Fig S5.tif]

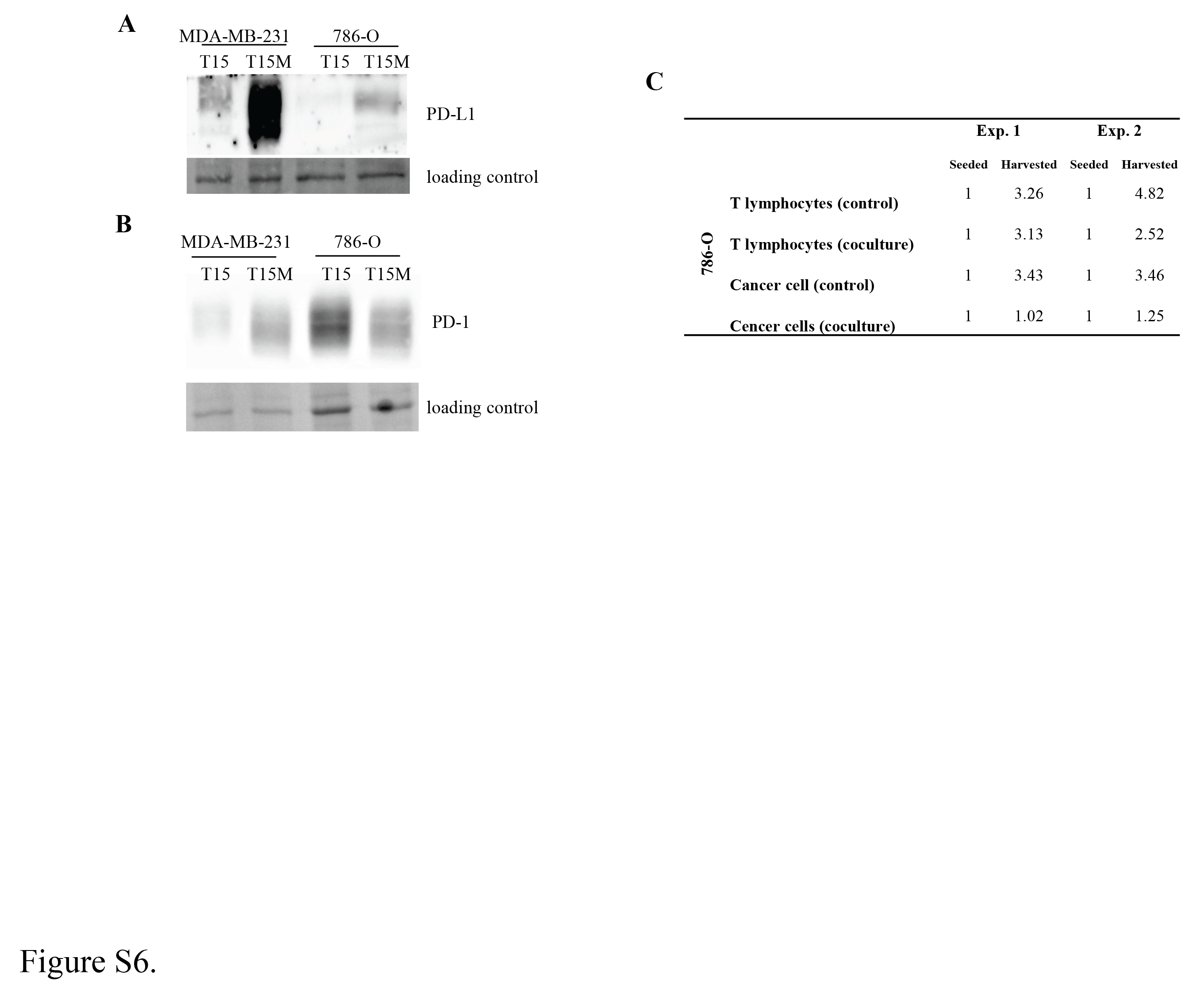

Supplement: Supplementary file 1 [file cancers-13-04148-s001.zip › Fig S6.tif]

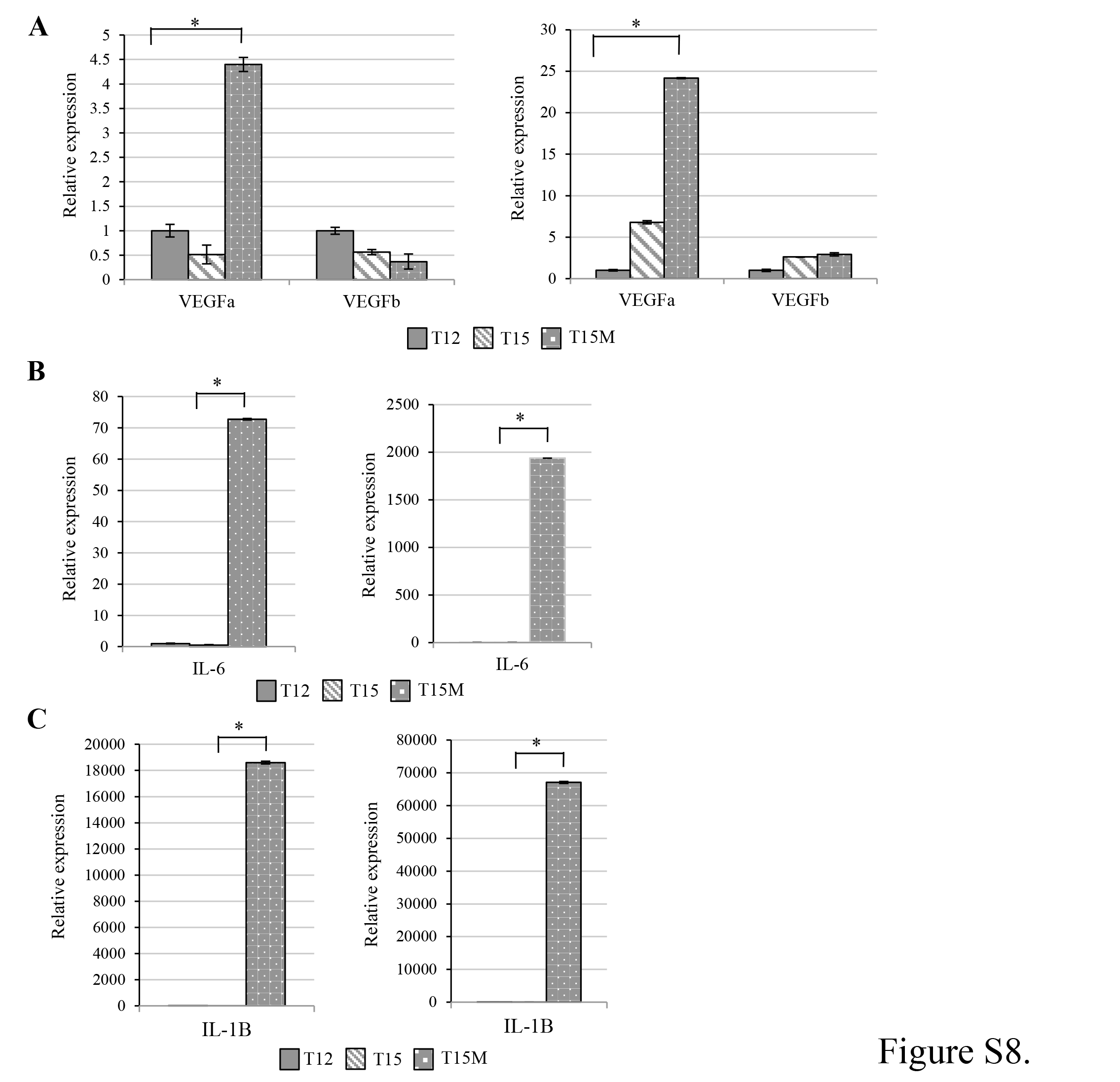

Supplement: Supplementary file 1 [file cancers-13-04148-s001.zip › Fig S7.tif]

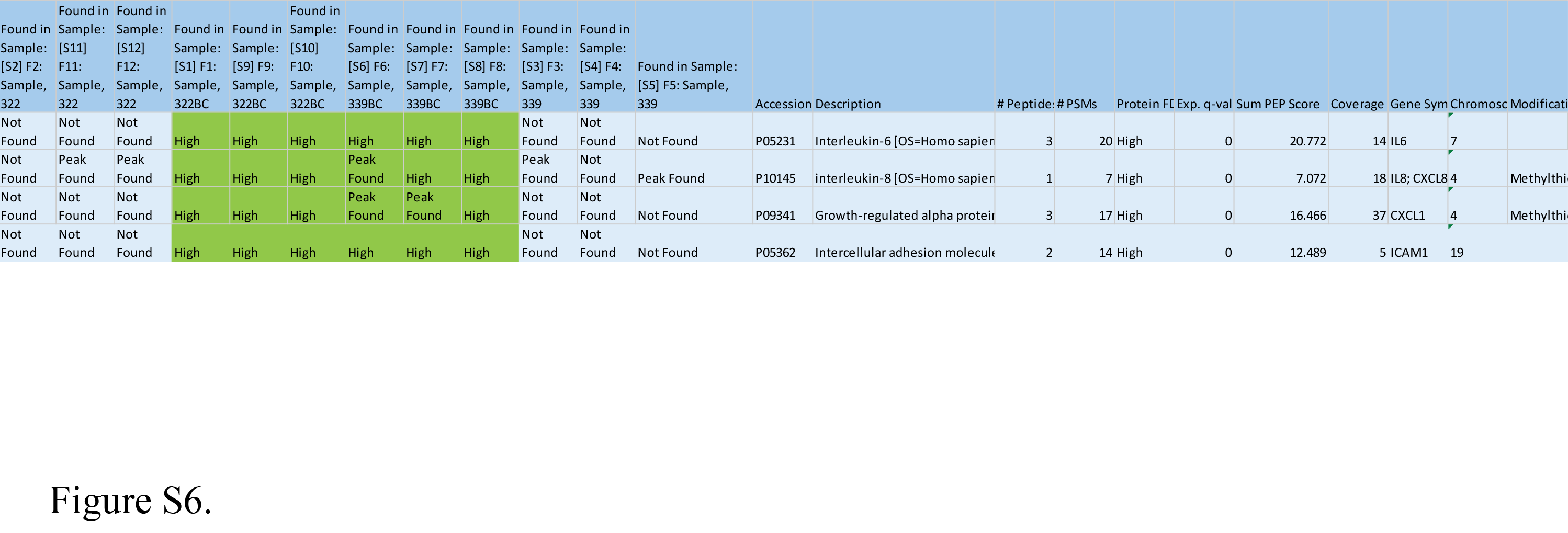

Supplement: Supplementary file 1 [file cancers-13-04148-s001.zip › Fig S8.tif]

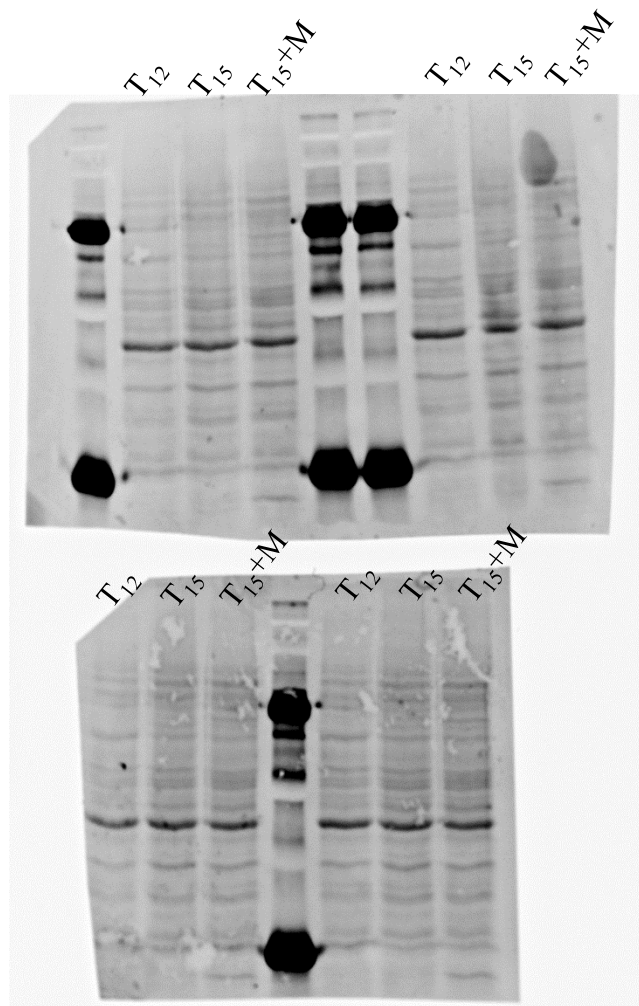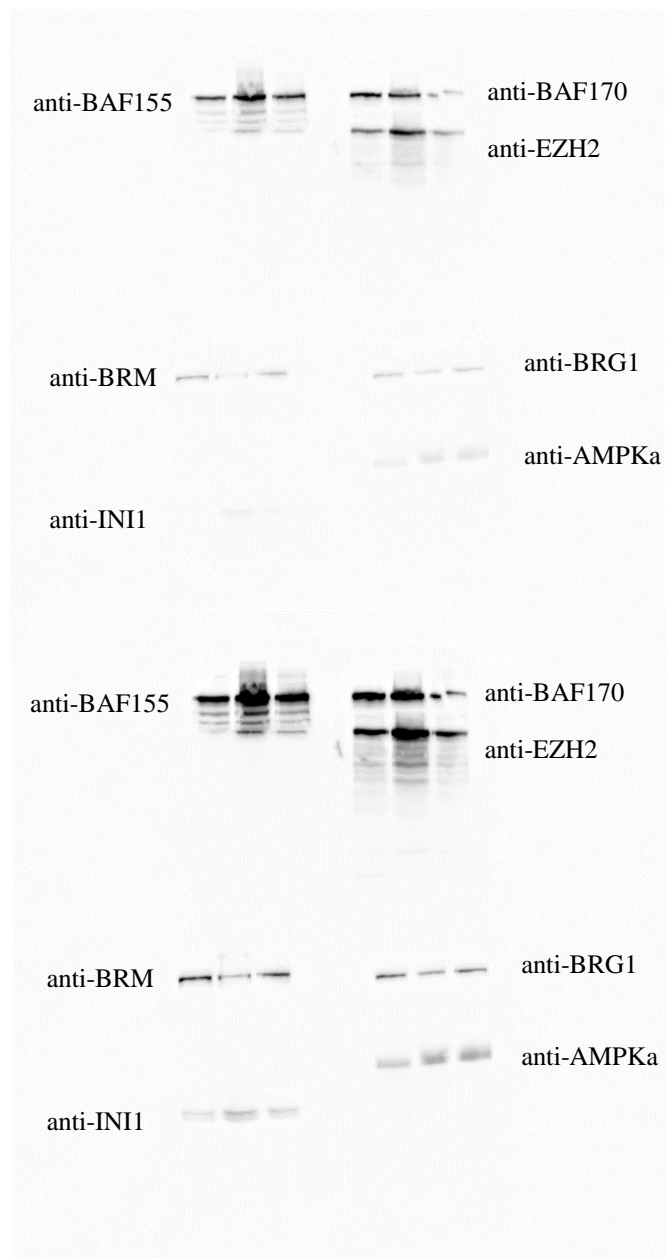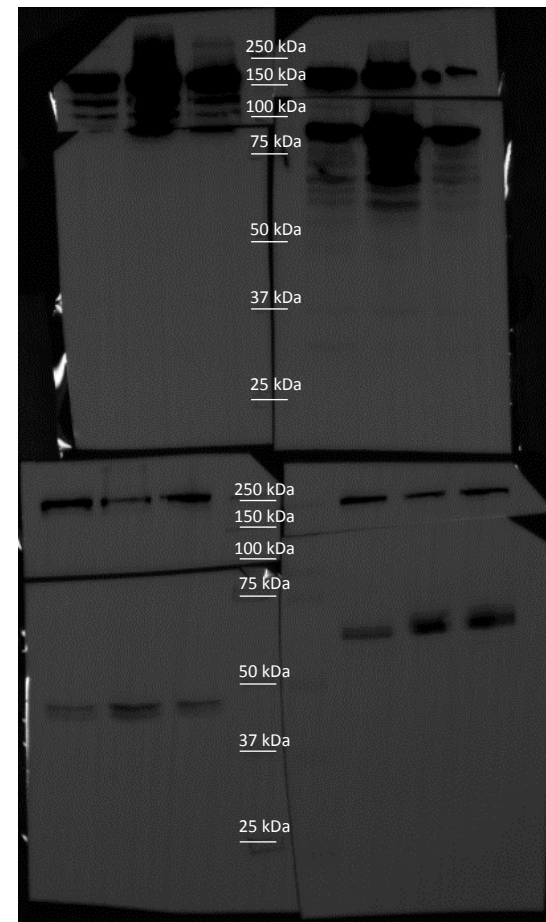

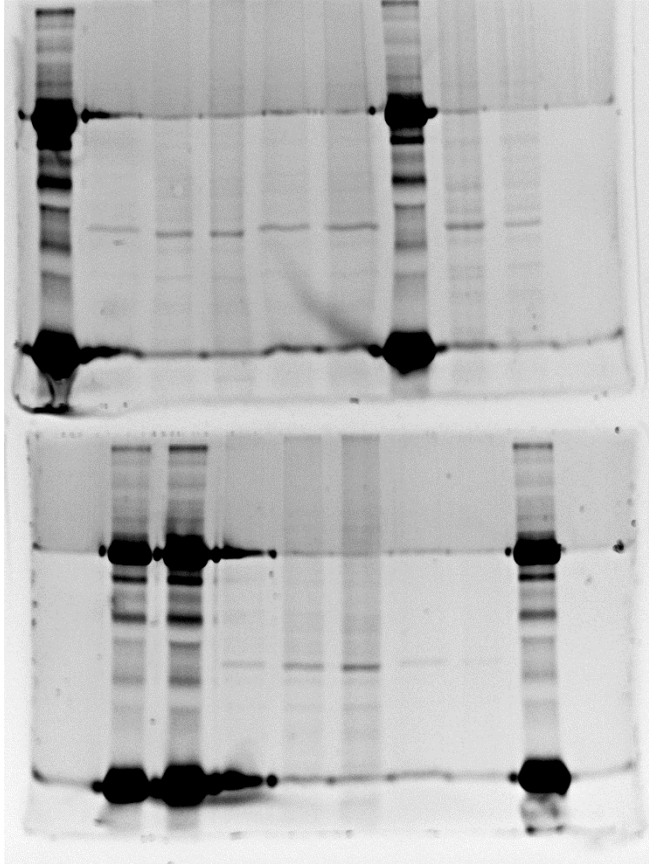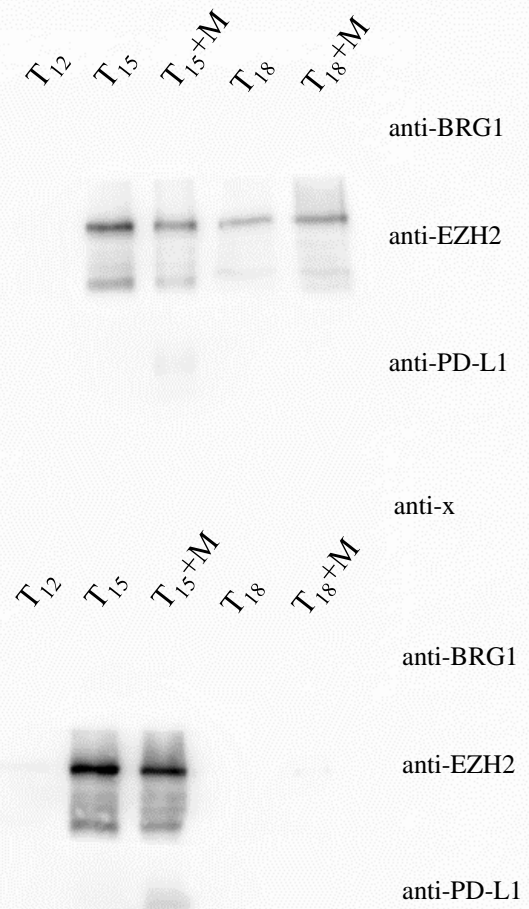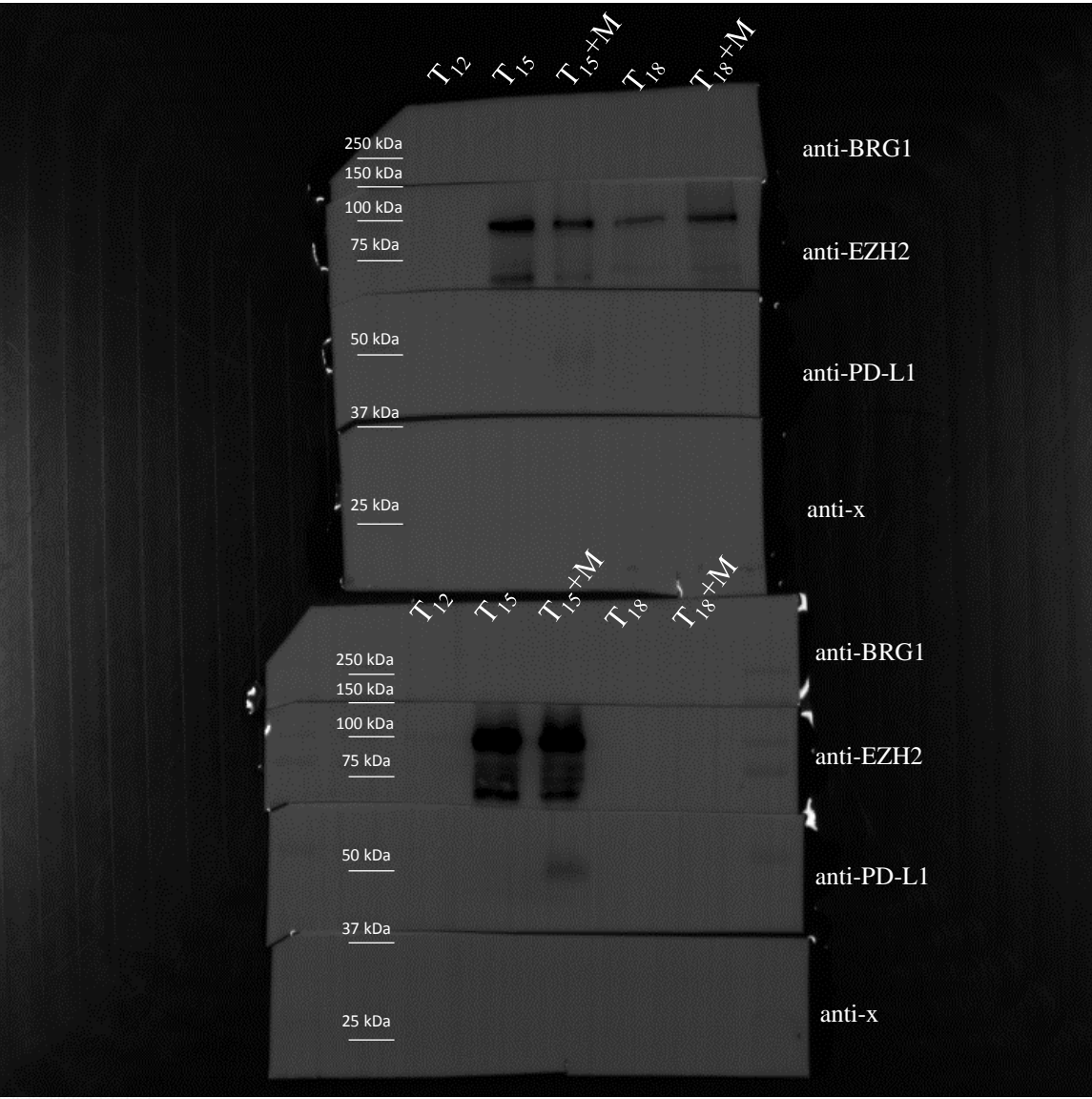

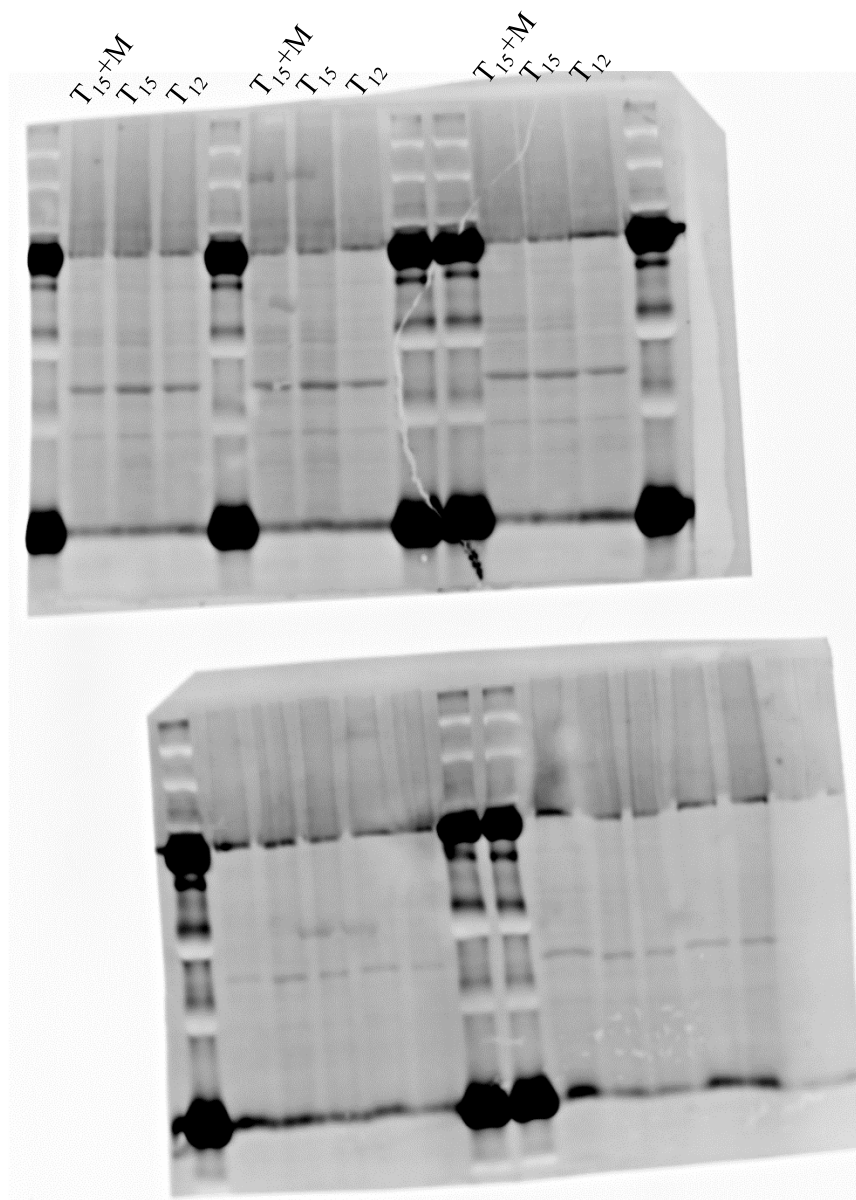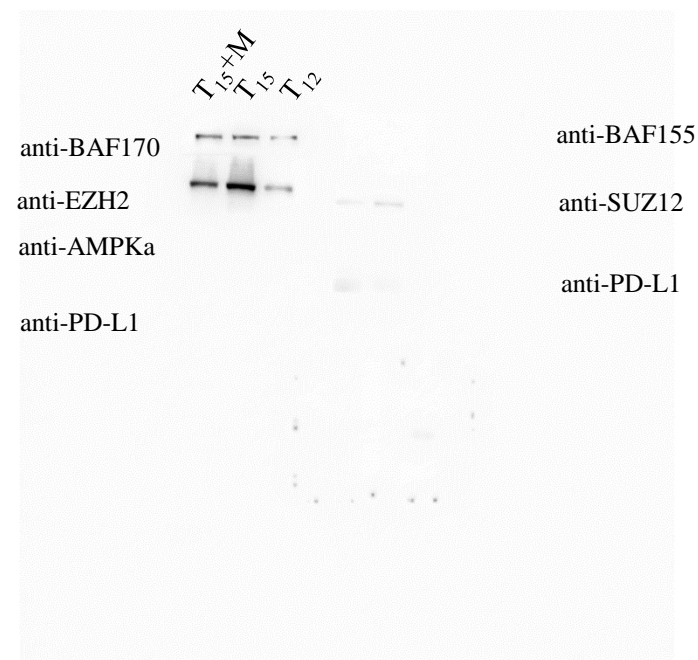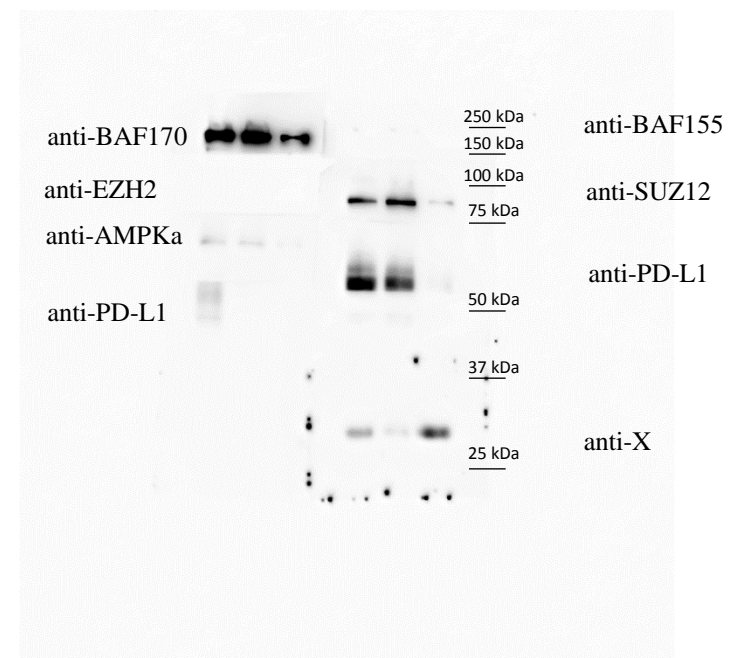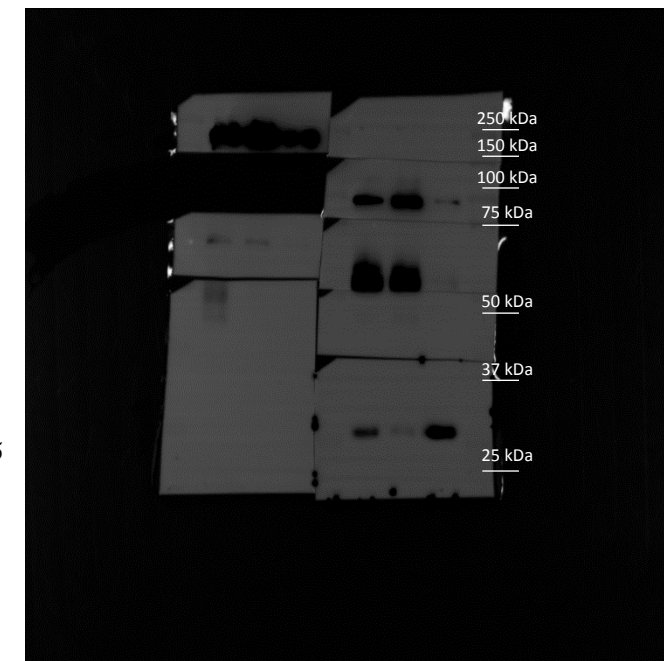

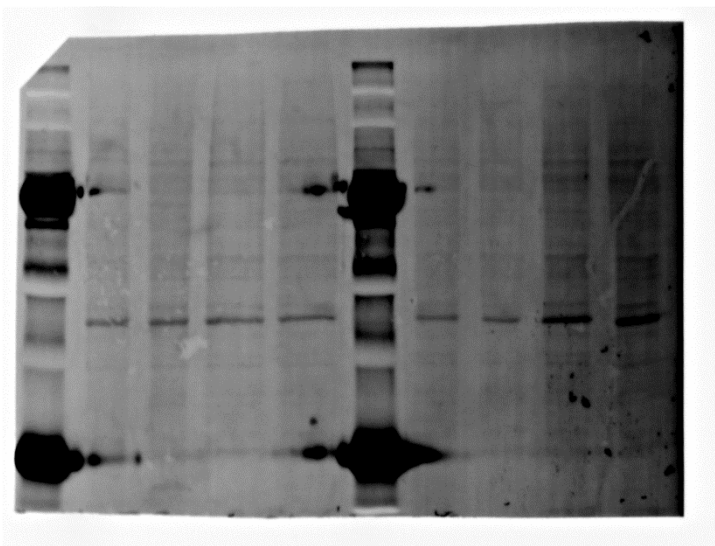

250 kDa

150 kDa

100 kDa

75 kDa

50 kDa

37 kDa

25 kDa

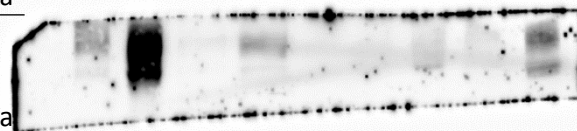

anti-PD-L1

318 i 319 WB

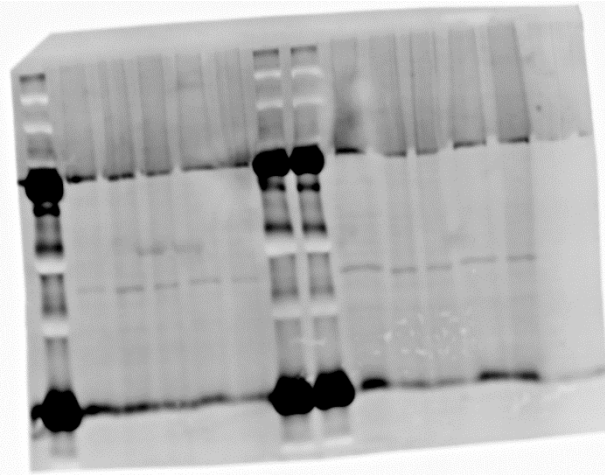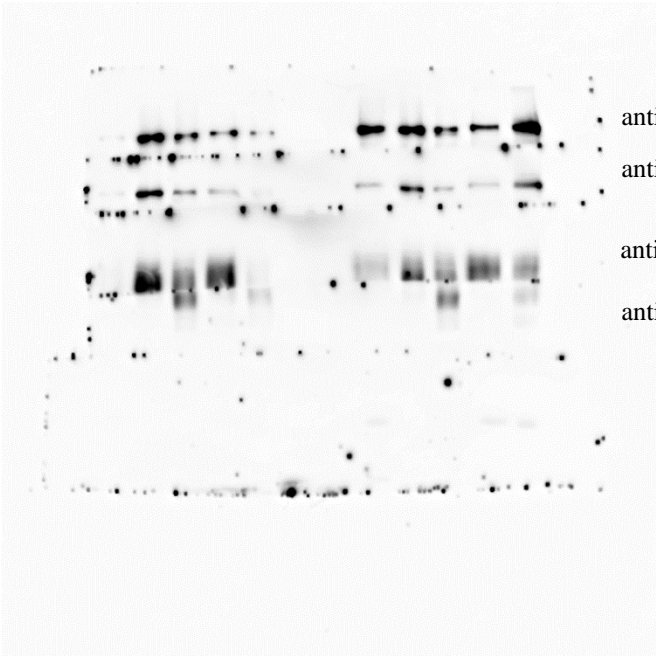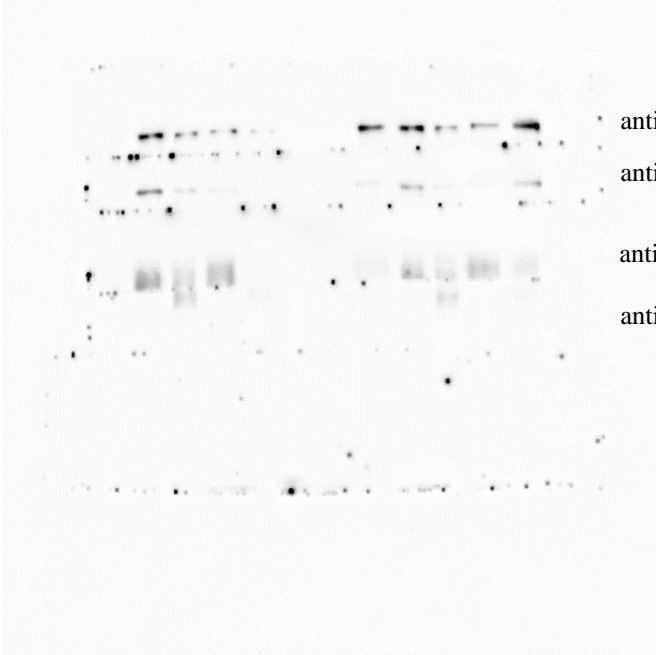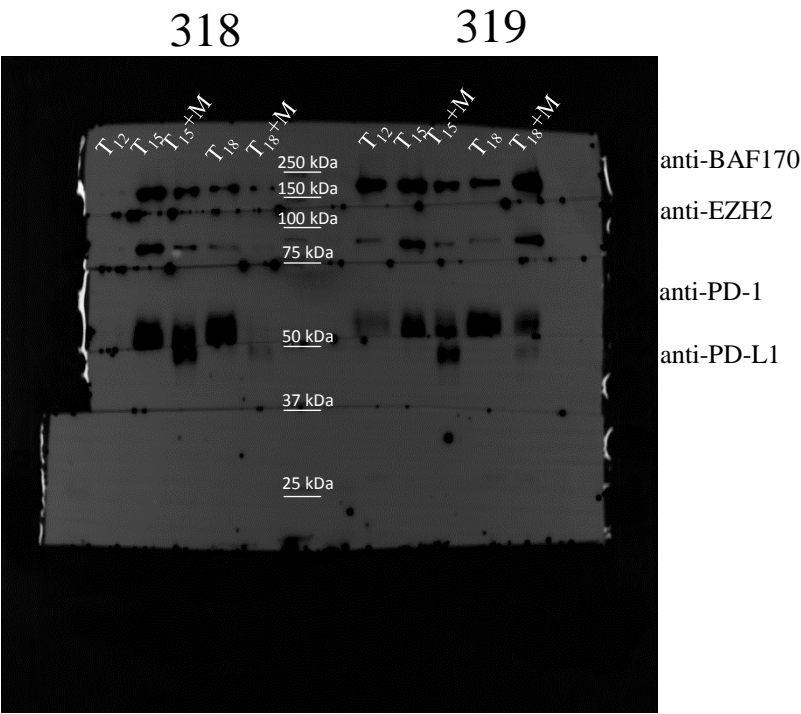

318 i 319 WB2

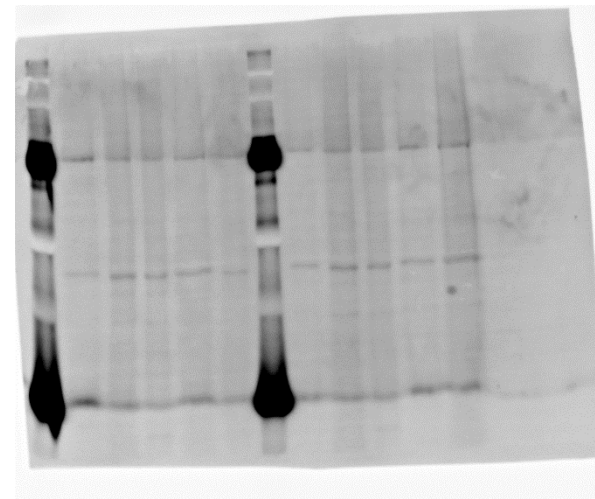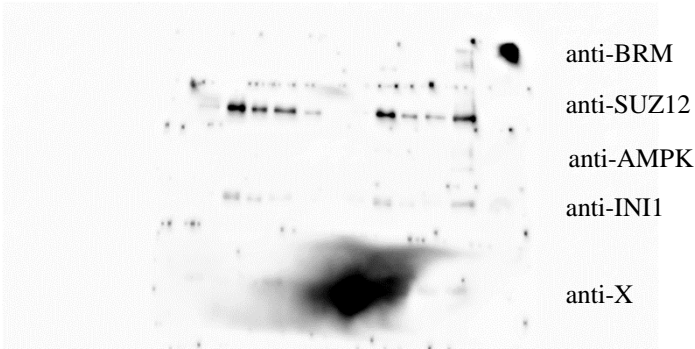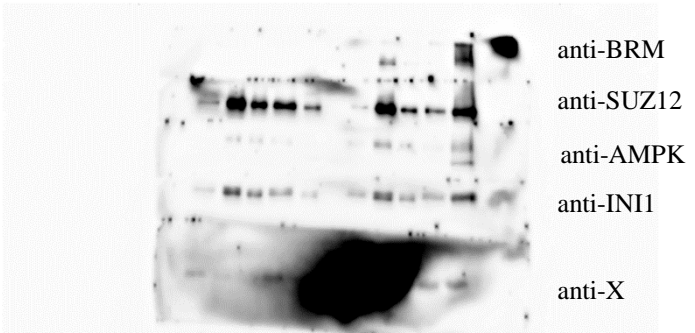

318 319

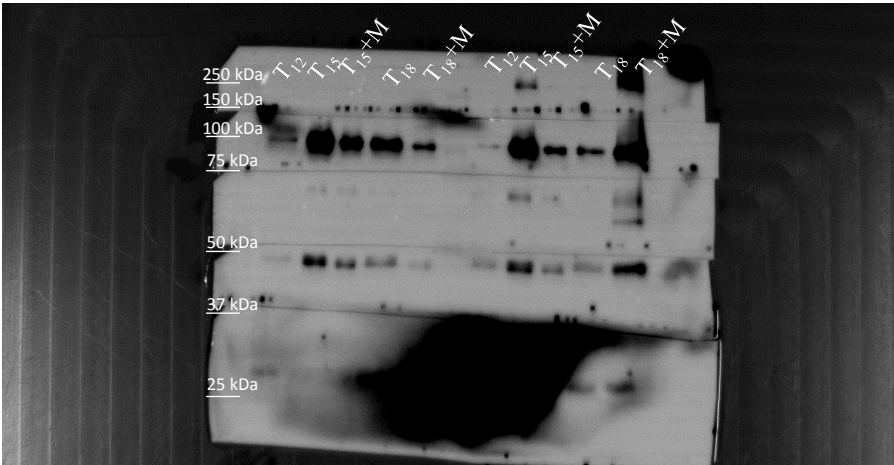

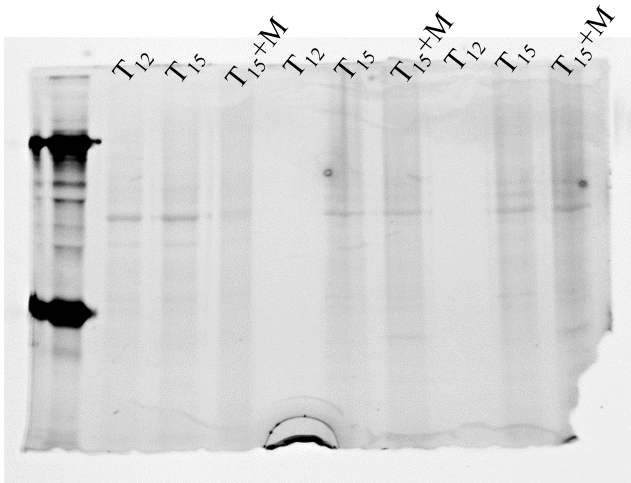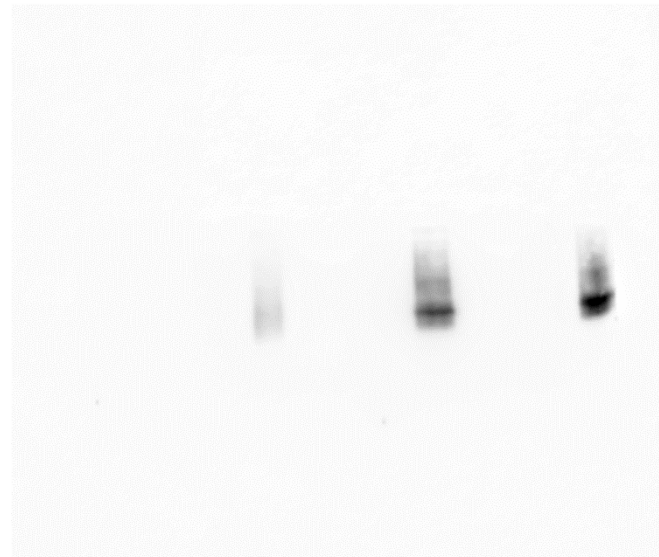

anti IL-1B

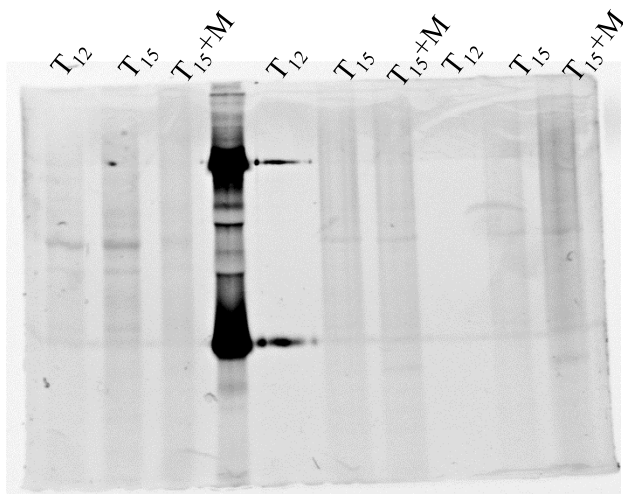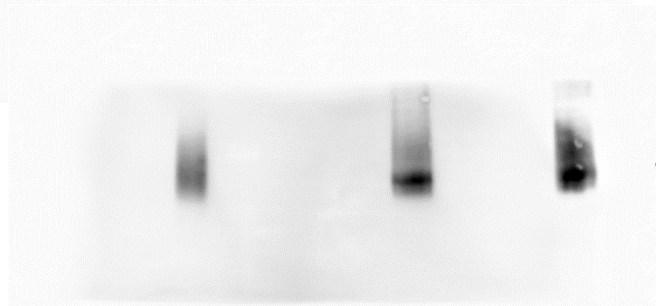

anti IL-6

Supplement: Supplementary file 1 [file cancers-13-04148-s001.zip › uncroped blots Jancewicz et al.pdf]
